# Geospatial Point-of-Care Testing Strategies for COVID-19 Resilience in Resource-Poor Settings: Rural Cambodia Field Study

**DOI:** 10.2196/47416

**Published:** 2024-08-27

**Authors:** Gerald Joseph Kost, Muyngim Eng, Amanullah Zadran

**Affiliations:** 1 Point-of-care Testing Center for Teaching and Research (POCT•CTR) School of Medicine University of California, Davis Davis, CA United States; 2 University of Phutisastra Phnom Penh Cambodia; 3 Public Health Sciences, POCT•CTR School of Medicine University of California, Davis Davis, CA United States

**Keywords:** Cambodia, COVID-19, diagnostic portals, mobile-testing van and clinic, molecular diagnostics, point-of-care testing, POCT, public health resilience, rapid antigen test, RAgT, SARS-CoV-2, Solano and Yolo counties, California

## Abstract

**Background:**

Point-of-care testing (POCT) generates intrinsically fast, inherently spatial, and immediately actionable results. Lessons learned in rural Cambodia and California create a framework for planning and mobilizing POCT with telehealth interventions. Timely diagnosis can help communities assess the spread of highly infectious diseases, mitigate outbreaks, and manage risks.

**Objective:**

The aims of this study were to identify the need for POCT in Cambodian border provinces during peak COVID-19 outbreaks and to quantify geospatial gaps in access to diagnostics during community lockdowns.

**Methods:**

Data sources comprised focus groups, interactive learners, webinar participants, online contacts, academic experts, public health experts, and officials who determined diagnostic needs and priorities in rural Cambodia during peak COVID-19 outbreaks. We analyzed geographic distances and transit times to testing in border provinces and assessed a high-risk province, Banteay Meanchey, where people crossed borders daily leading to disease spread. We strategized access to rapid antigen testing and molecular diagnostics in the aforementioned province and applied mobile-testing experience among the impacted population.

**Results:**

COVID-19 outbreaks were difficult to manage in rural and isolated areas where diagnostics were insufficient to meet needs. The median transit time from border provinces (n=17) to testing sites was 73 (range 1-494) minutes, and in the high-risk Banteay Meanchey Province (n=9 districts), this transit time was 90 (range 10-150) minutes. Within border provinces, maximum versus minimum distances and access times for testing differed significantly (*P*<.001). Pareto plots revealed geospatial gaps in access to testing for people who are not centrally located. At the time of epidemic peaks in Southeast Asia, mathematical analyses showed that only one available rapid antigen test met the World Health Organization requirement of sensitivity >80%. We observed that in rural Solano and Yolo counties, California, vending machines and public libraries dispensing free COVID-19 test kits 24-7 improved public access to diagnostics. Mobile-testing vans equipped with COVID-19 antigen, reverse transcription polymerase chain reaction, and multiplex influenza A/B testing proved useful for differential diagnosis, public awareness, travel certifications, and telehealth treatment.

**Conclusions:**

Rural diagnostic portals implemented in California demonstrated a feasible public health strategy for Cambodia. Automated dispensers and mobile POCT can respond to COVID-19 case surges and enhance preparedness. Point-of-need planning can enhance resilience and assure spatial justice. Public health assets should include higher-quality, lower-cost, readily accessible, and user-friendly POCT, such as self-testing for diagnosis, home molecular tests, distributed border detection for surveillance, and mobile diagnostics vans for quick telehealth treatment. High-risk settings will benefit from the synthesis of geospatially optimized POCT, automated 24-7 test access, and timely diagnosis of asymptomatic and symptomatic patients at points of need now, during new outbreaks, and in future pandemics.

## Introduction

Our goals are to mitigate outbreaks, enhance standards of care, and improve public health resilience toward the COVID-19 pandemic and highly infectious threats that could arise in the future. The research objectives were (1) to identify the need for point-of-care testing (POCT) in limited-resource Cambodian border provinces during peak outbreaks of COVID-19 and (2) to quantify geospatial gaps in access to COVID-19 diagnostics.

In parallel, insights gleaned from the automated distribution of diagnostics and mobile van testing in the rural Solano and Yolo counties in California helped us frame recommendations for limited-resource settings. Mobile clinics and testing for COVID-19 have proven effective in the United States [1-3] and can be used in association with contact tracing [4] and other public health strategies [5-8] to increase testing among underserved and vulnerable populations, such as people in nursing homes [9]. In essence, mobile testing and expanded diagnostic portals improve community accessibility to rapid medical care, an important advantage for public health responses to highly infectious diseases [10].

We gathered field data from rural Cambodian border provinces during periods of widespread outbreaks, border closures, and community lockdowns peaking in 2021 to understand practices that enhance responses to the COVID-19 pandemic and public health resilience in remote communities. Cambodia, which has a population of 17.2 million people, performed more than 3 million COVID-19 diagnostic tests, reported over 3000 deaths [11], and still experiences a few active cases. American deaths indirectly related to COVID-19 are approximately 1.2 million, while approximately 1.22 million direct COVID-19 fatalities bring the total loss of life to >2.4 million [11-14]. In rural Cambodian border provinces, the COVID-19 pandemic may have disproportionately impacted vulnerable individuals and those with pre-existing diseases [15-18] who lacked fast access to diagnostics.

Documenting COVID-19 testing needs in Cambodia during peak outbreaks and addressing them through geospatial analysis and shared experience in rural Solano and Yolo counties of California supported the goal of enhancing the standards of care for those with highly infectious diseases. Therefore, to augment resilience using point-of-care (POC) diagnostics, we synthesized transpacific strategies to meet the identified needs of the targeted populations, fill geospatial gaps among the people of rural Cambodian border provinces, and anticipate future public health crises.

## Methods

### Needs Assessment in Cambodia: Data Collection

To accomplish our first research objective, we performed POCT needs assessment in Cambodia on the basis of prior field research in limited-resource settings [19-24] and methods codified by Point-of-Care Technologies Centers and Research Network investigators and funded by the US National Institute of Biomedical Imaging and Bioengineering at the National Institutes of Health [25-27]. Considering the widespread reach of COVID-19, we surveyed academicians, professionals, business operators and employees, and community laypersons to assess the potential for use of rapid antigen tests (RagTs), self-testing and mobile diagnostics, and the suitability of travel to reverse transcriptase polymerase chain reaction (RT-PCR)–testing referral sites.

We generated a questionnaire in English and the Khmer language (available in Multimedia Appendix 1) to rapidly gather data related to the challenging conditions of episodic quarantines, red zones of high risk, and prolonged lockdowns. We used the structured questionnaire to systematically and rapidly identify and collate needs through meetings, university exchanges, webinar discussions, lecture follow-up and feedback, Zoom sessions, facility inspections, field interviews, and a focus group (13 participants). Overall, 137 participants contributed to the assessment survey and compilation of needs.

Contributing survey respondents comprised (1) public health, clinical laboratory, field testing, and medical personnel including professors, physicians, nurses, pharmacists, a public health analyst, medical technologists, a quality control supervisor, emergency medical system operators, and private laboratory staff; (2) a civil servant, project manager, and biologist; (3) professional leaders comprising the President of the Cambodian National Association of Medical Technologists, the faculty of the University of Puthisastra, and the director, workers, and students from the Ministry of Health National Public Health Laboratory and the National Institute of Public Health in Phnom Penh; (4) road checkpoint officials in provinces, tourist agents, community workers, transportation staff, hotel personnel, Angkor Wat employees in Siem Reap Province, and personnel working in nongovernment organizations; and (5) the Phnom Penh City pandemic committee that monitored outbreaks and community police officers who enforced quarantines.

We augmented respondent data by making numerous telephone calls; by engaging manufacturers of tests (in Phnom Penh, Singapore, South Korea, and Thailand); by monitoring commercial sales status in Cambodia; through email correspondence; and by merging the data provided by local health care experts in Banteay Meanchey, other border provinces, and Phnom Penh. The residents and family members in Siem Reap Province—a site of high rural infection rates that shares heavily used transportation routes with the Banteay Meanchey border province to the west—were queried as well as Cambodian workers, some of whom were stranded when Thailand, under duress from increasing contagion, unilaterally closed border gateways.

### Geospatial Analysis of Access to Diagnostics in Cambodian Border Provinces: Variables and Measurements

To accomplish our second research objective, we used geospatial science techniques innovated for limited-resource settings by Ferguson et al [28-30] and Kost [31,32]. This was done to metricize distances from the main villages of each district within the Cambodian border provinces to COVID-19 testing sites at province referral hospitals. Transit times were calculated by dividing the distances traveled by the speed limit of 30 km/h for motorcycles in towns. In general, rural residents did not have access to automobiles or buses for routine transport.

**Figure 1:**
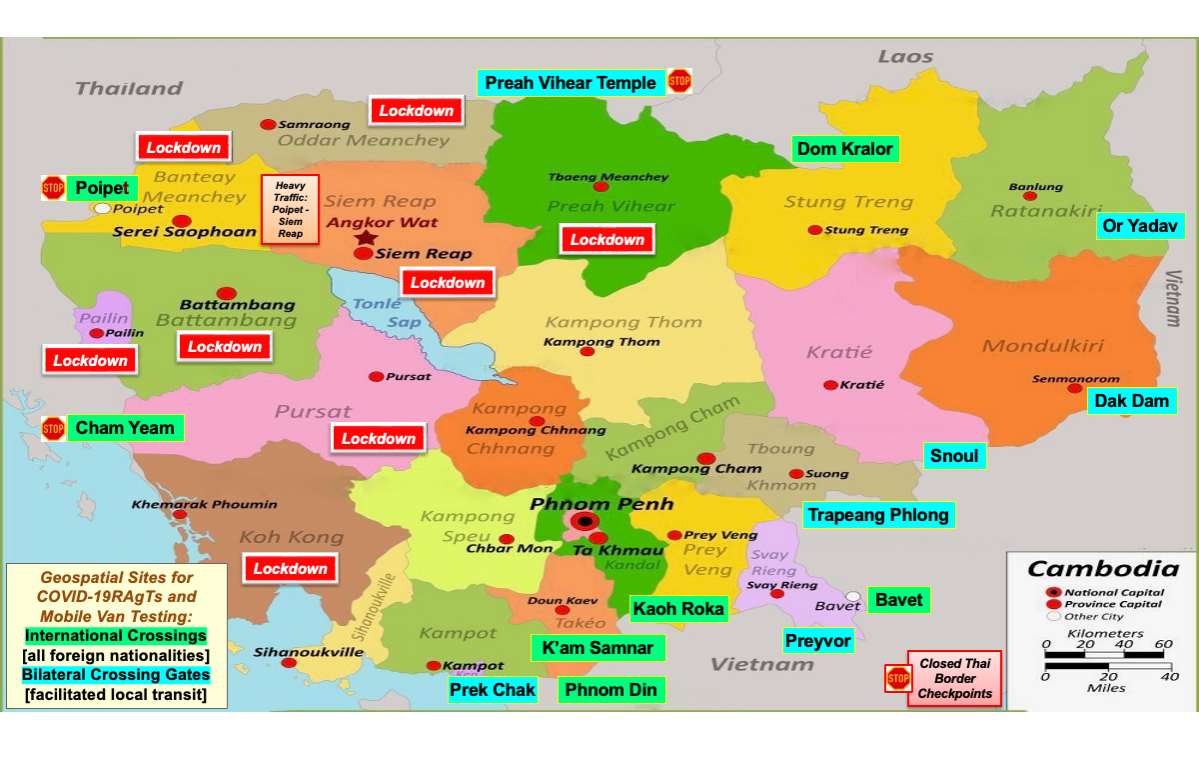
Lockdowns, border crossing sites, and recommended COVID-19 testing locations during the peak epidemic in Cambodia with geospatial sites for international crossings and bilateral crossing gates. Provinces were locked down, and borders closed in Cambodia during COVID-19 surges from April to August 2021. Banteay Meanchey Province experienced high contagion caused by human border traffic to and from Thailand, which unilaterally shut down Poay Paet (Poipet) border crossings and stranded Cambodian migrant workers on the Thai side. Relatives who were out of work and who escaped Phnom Penh red-zone lockdowns returned home to villages and precipitated family outbreaks in rural Siem Reap Province, which was locked down.

Figure 1 identifies provinces, international borders, bilateral crossing gates, border closures, and lockdowns. Physical locations were based on data obtained from web-based open-source mapping software (Cambodia Covid Restrictions Map 2021 MapItKH [33], OnTheWorldMap [34]), hardcopy Cambodia and Thailand maps, web resources [National Institute of Public Health, *Phnom Penh Post,* *Khmer Times*, digital libraries], and literature searches (PubMed, Google). Population census, national health care data, and public health reports (Open Development Cambodia [35]) helped determine public accessibility to local COVID-19 testing sites.

### Statistical Analysis

Province data are reported as median distance and median time to testing for each province in descending-value Pareto plots, which also identify 20% thresholds for cumulative distance. Differences were assessed using the Kruskal-Wallis test followed by pairwise analysis (using the Mann-Whitney *U* test). Paired differences (maxima minus minima) were analyzed using the Wilcoxon signed rank test. A value of *P*<.05 was considered significant. Data were not distributed normally (Shapiro-Wilk test). However, for Banteay Meanchey Province, we reported the median (range) and mean (SD) distances to testing and the transit time to document distribution skewness.

### Performance Evaluation

Popular COVID-19 RAgTs in Southeast Asia at the time of the peak outbreaks in Cambodia included the Roche Diagnostics SARS-CoV-2 RAgT and the Abbott Panbio RAgT. Mathematical analyses [10,36-39] were conducted in parallel with field research in Cambodia to (1) determine if these two tests met the World Health Organization (WHO) criterion for sensitivity >80% (median) in clinical evaluations, (2) characterize performance patterns and their dependence on prevalence, and (3) support strategic recommendations in the discussion. Tables S1 and S2 in Multimedia Appendix 1 tabulate the clinical evaluations, Figures S1 and S2 in Multimedia Appendix 1 compare performance plots, and Table S3 in Multimedia Appendix 1 lists Bayesian formulas derived to graph performance.

### Automated COVID-19 Test Dispensers

To improve accessibility to diagnostics, a COVID-19 RAgT vending machine was placed outside the library in Esparto, the first site in California to implement automated, 24-7, free public access to testing, which has expanded now to include free masks, tick and insect repellent, male and female condoms, and naloxone. Esparto is a small rural community of 4009 people near the western foothills of Yolo County. Additional county sites were established outside the Davis Library, West Sacramento Community Center, Winters City Hall, and La Superior Market in Woodland.

One could obtain as many test kits (eg, Flowflex; ACON LABS; Bio-Self, Bioteke USA) as needed by simply tapping a key on the vending machine. There was no need for identification, registration, or follow-up. Asymptomatic individuals could also obtain free RAgT test kits (InteliSwab; Orasure Technologies) from staff inside county public libraries. Local access continues through 2024; the US government has ended free RAgT access online, having distributed over 900 million test kits, but still provides them to uninsured individuals and underserved communities. Papers by Kost [37,40] illustrate storyboards of the self-testing process steps. The National Institutes of Biomedical Imaging and Bioengineering (NIH Radx Tech Initiative) implemented a “Make My Test Count” website for anonymous reporting of self-testing results [41].

### Ethical Considerations

The Ethics Committee at the University of Puthisastra, one of the Fulbright Scholar academic sponsors (for author GJK) in Phnom Penh, approved this research (IR010; May 24, 2021). No patient records were accessed. No personal information was recorded. Data were collected from January 2021 through June 2024.

## Results

### Diagnostic Needs in Rural Cambodian Border Provinces

Respondents in rural Cambodian provinces identified needs comprising rapid on-time COVID-19 screening with high-quality COVID-19 antigen tests (top priority) that minimize false negatives, increased testing of people crossing borders and moving in and out of contagion zones, and increased accessibility of RT-PCR testing. They requested mobile testing for people living in immigration sites; at drive-throughs; in communities (families, villages, markets, and at pagodas); at emergency rooms; at nursing homes; and in airports. Several people noted the need for careful maintenance of biosafety, quality control, and supply chains. There was little discussion of antibody testing or the need for it. The responses allowed us to summarize technical diagnostic needs as follows.

Technical needs identified by the respondents included portable cartridge- or cassette-based molecular diagnostic instruments characterized with few processing steps to improve the ease of testing and assure biosafety in community settings, especially remote rural areas; adequate personal protective equipment and district hospital rapid response to accelerate treatment and preoperative diagnosis; operator training, quality monitoring, and performance evaluations; improved public education to encourage the use of POCT; specimen handling protocols in which onsite testing was not possible and specimens must be sent out; widespread self-testing to help mitigate outbreaks and stimulate economic recovery; and SARS-CoV-2 variant sequencing.

On the western border with Thailand, the three-province cluster of Banteay Meanchey, Battambang, and Siem Reap, plus the relatively isolated Koh Kong Province (identified in Figure 1), as well as Pre Veng and Takeo provinces on the eastern border with Vietnam, were named by the respondents as top priorities for public health intervention. Delta and then Omicron variants were detected in Thai transient workers in Battambang and other border regions. Oddar Meancheay (remote northwest) lacked testing resources despite increasing contagion. Other high-risk regions and their risk factors identified encompassed Kampong Cham (factories and urban spread); Kampong Speu (prison); Kandal (casinos, factories, prison, and urban spread); Mondulkiri; Phnom Penh (high caseload and lockdown districts); Sihanoukville (casinos); Stung Treng (remote northeast); Svey Rieng (factories and high contact-tracing alerts); and Tboung Khmom provinces.

Respondents emphasized the need for public release of testing data, clearer citizen instructions for mandatory testing upon entry to a province, communication of testing site locations, formal evaluation of imported test kits, and free RAgTs with easily interpreted results. One respondent noted that 3 to 5 days was too long to wait for RT-PCR results. Another said not to test vaccinated people unless symptomatic and recommended that RT-PCR testing be placed at or near borders to lower testing costs. All respondents wanted root-cause solutions that would decrease the spread of COVID-19 in areas of high population density.

Respondents stated that the goals of national policy and guidelines for COVID-19 should include public health strategies and recommendations for test-positive people and their families; systematic procedures for contact tracing, isolation, and self-quarantine; testing throughout Cambodia to document positivity rates; education regarding the differences in RAgTs, molecular diagnostics, and antibody tests; collaboration of bioengineers and informatics specialists; diagnostic criteria for advanced medical care and hospitalization; accessibility of diagnostics to vulnerable populations; and ultimately, accelerated evidence-based treatment of infected patients.

### Geospatial Access to Diagnostics in Cambodian Border Provinces

**Figure 2:**
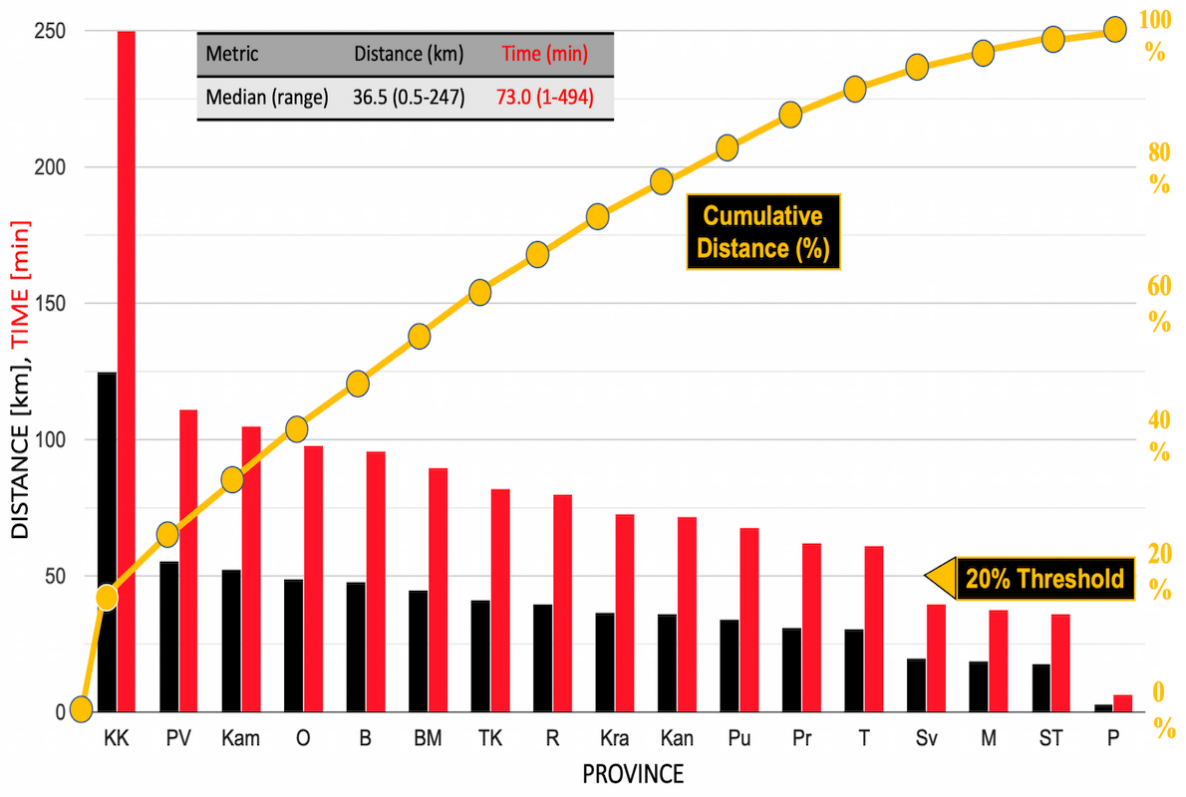
A Pareto plot of distances and transit times to COVID-19 testing locations in Cambodian border provinces. The curve representing cumulative distance shows that Koh Kong Province nearly exceeds the Pareto 20% threshold and therefore, is most in need of locally available diagnostics. B: Battambang; BM: Banteay Meanchey; Kam: Kampot; Kan: Kandal; KK: Koh Kong; Kra: Kratié; M: Mondulkiri; O: Oddar Meanchey; P: Pailin; Pr: Prey Veng; Pu: Pursat; PV: Preah Vihear; R: Ratanakiri; ST: Stung Treng; Sv: Svay Rieng; T: Takeo; TK: Tboung Khmom.

Figure 2 displays the Pareto plot for distance and time to testing in 17 border provinces (Table 1). A Pareto plot displays data in the order of descending magnitude along the horizontal axis. The legend identifies the provinces (and their locations in Figure 1). The median distance from province districts to testing was 36.5 (range 0.5-247) km, and the median transit time was 73 (1-494) minutes. Within provinces, the median of the paired differences in maximum versus minimum distances to testing was 70 km (*P*<.001); for transit time, this value is 2.3 hours, demonstrating uneven access to COVID-19 diagnostics.

Koh Kong Province (Figure 1), which has a long undeveloped coastline and a mountainous, forested, and largely inaccessible interior, had one of the longest distances to testing of 125 km and prolonged one-way transit time of 4.17 hours. A district in Stung Treng Province had the longest distance (247 km) and the most prolonged time to reach a testing site (8 hours, 14 minutes).

**Figure 3:**
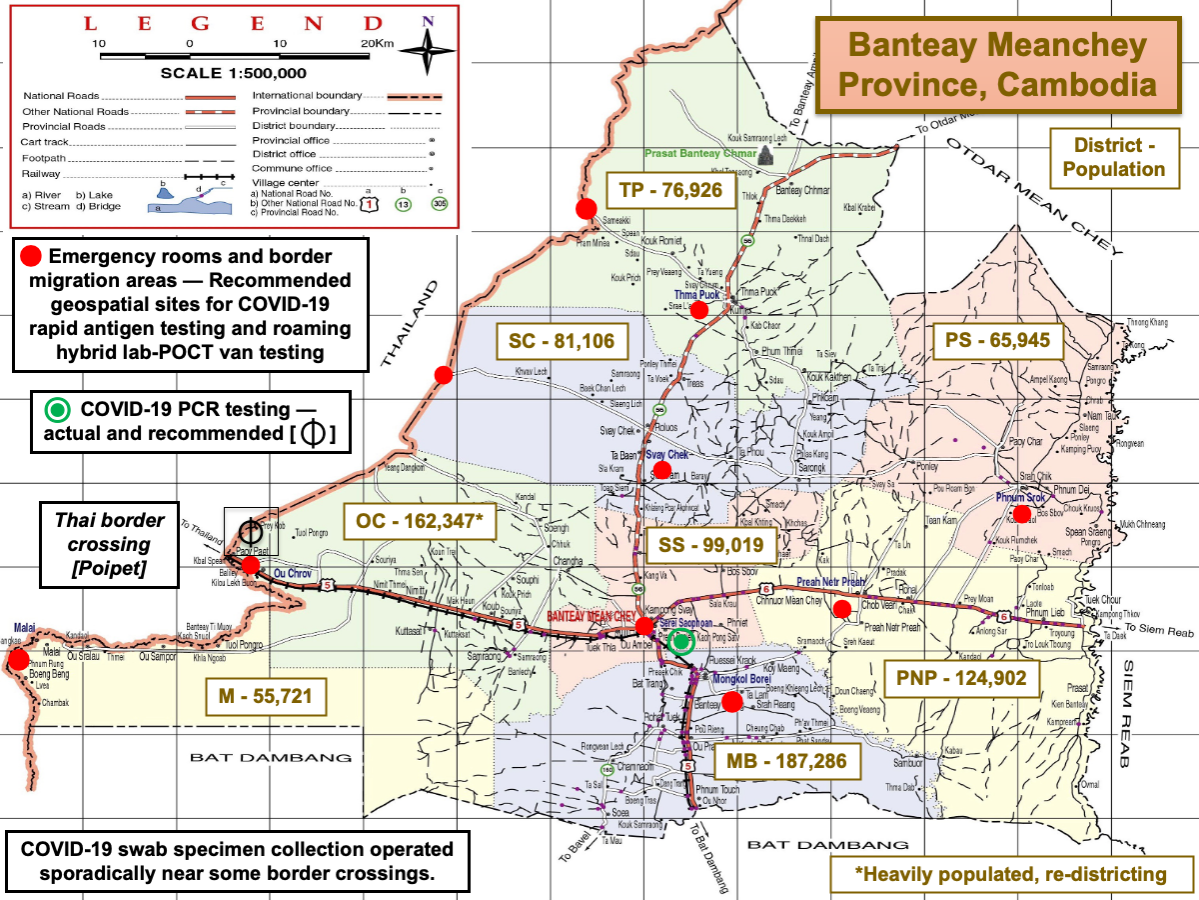
Recommended geospatial sites for COVID-19 rapid antigen testing and mobile van testing in rural Banteay Meanchey Province during COVID-19 surges. KPP: Krong Paoy Paet; M: Malai; MB: Monkol Borei; OC: Ou Chrov; POCT: point-of-care testing; PNP: Preah Netr Preah; PS: Phnum Srok; SC: Svay Chek; SS: Serie Saophoan; TP: Thma Puok.

Figure 3 presents the Banteay Meanchey Province on the eastern border of Thailand. This province is divided into 9 districts with populations shown on the map. Table 2 details district names; main villages; and demographic, geographic, and risk features at the time of COVID-19 surge. During the 2021 peak, RAgTs were not available to the public. Among 72 public health facilities, 1 hospital offered RT-PCR testing (Cambodia-Japan Friendship Mongkul Borey Provincial Hospital). Two others (Serey Sorphorn and Poipet Referral Hospitals) offered RAgTs.

**Figure 4:**
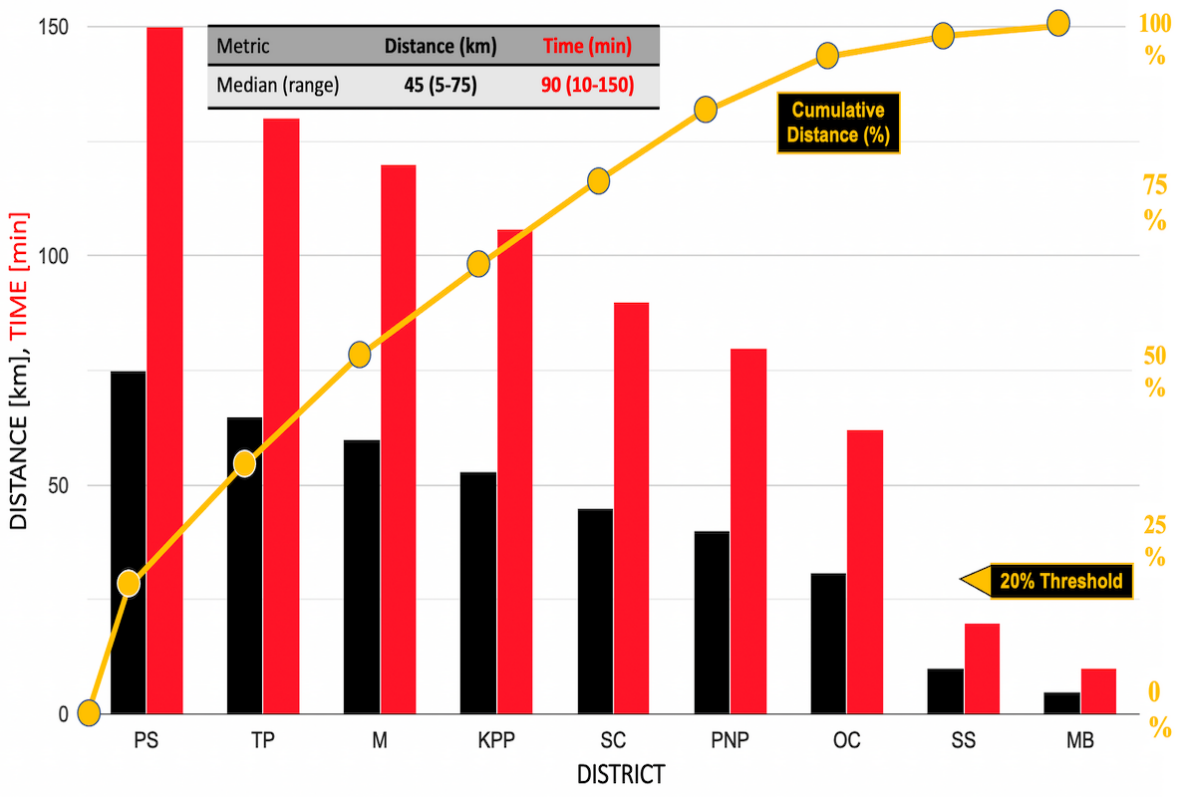
Pareto plot of distances and transit times to COVID-19 testing locations in the districts of Banteay Meanchey Province, which have high community populations and risk for COVID-19. KPP: Krong Paoy Paet; M: Malai; MB: Monkol Borei; OC: Ou Chrov; PNP: Preah Netr Preah; PS: Phnum Srok; SC: Svay Chek; SS: Serie Saophoan; TP: Thma Puok.

Figure 4 shows the Pareto plot for the Banteay Meanchey districts. The median distance to testing was 45 (range 5-75) km. In descending order, transit times ranged from 150 to 10 minutes. Phnum Srok District, an isolated mountainous area in which people face difficulties and delayed emergency care access in the northwest corner of the province, had the maximum distance (75 km) and transit time (2.5 hours; Table 2). Median transit time was 90 (range 10-150) minutes. The mean was 42.7 (SD 23.9) km.

**Table 1:** Geospatial analysis of Cambodian border provinces: median distance and time from main districts to COVID-19 testing sites within each border province during the COVID-19 pandemic (N=17).

Border provinces (Pareto plot abbreviations)		Distance to test (km)					∆ Distance (km; maximum – minimum)			Transit time (min)			∆ Time (min; maximum –minimum)
Koh Kong (KK)		125					141.4			250			282.8
Preah Vihear (PV)		55.5					81			111			162
Kampot (Kam)		52.5					75			105			150
Oddar Meanchey (O)		49					87			98			174
Battambang (B)		48					109			96			218
Banteay Meanchey (BM)		45					70			90			140
Tboung Khmom (TK)		41.15					66.2			82.3			132.4
Ratanakiri (R)		40					65			80			130
Kratié (Kra)		36.5					72			73			144
Kandal (Kan)		36					46			72			92
Pursat (Pu)		34					99			68			198
Prey Veng (Pr)		31					69			62			138
Takeo (T)		30.5					33			61			66
Svay Rieng (Sv)		20					38			40			76
Mondulkiri (M)		19					58			38			116
Stung Treng (ST)		18					234.9			36			469.8
Pailin (P)		3.25					5.5			6.5			11
**Statistics**													
	Overall median (range)		36.5 (0.5-247.0)		70 (5.5-234.9)			73.0 (1-494)			140 (11.0-469.8)		
	*P* value^a^		—^b^		<.001			—			<.001		
Siem Reap^c^				25.5		—			51			—	

^a^Wilcoxon signed ranks test was used to determine the *P* value.

^b^Not applicable.

^c^This province (not included in the median above), located inland on the eastern border of Banteay Meanchey and listed below, was visited to collect field research data about its high tourist volume, heavy rural contagion as workers returned home infected from Phnom Penh, and COVID-19 screening at traffic checkpoints, which author GJK experienced. Angkor Wat is located in the rural region north of the city.

Border provinces were locked down from July through August 2021 (Figure 1). A highway from Thailand passing through Banteay Meanchey Province into Siem Reap City, one of the largest cities in Cambodia, witnessed heavy traffic on normal days, which was not the scenario during lockdowns. The lockdowns isolated Siem Reap Province where the median time to a testing site was 51 (mean 85.8, SD 74.4, range 10-230) minutes. In general, in rural provinces, isolated lockdown regions had limited hospital beds, critical care facilities, respiratory ventilators, laboratory resources, diagnostic instruments, and personal protective equipment, which impaired health care access to treatment for rural populations.

**Table 2:** Geospatial analysis of rural Banteay Meanchey Province, Cambodia: distance, time, demographics, and COVID-19 risk features during peak outbreaks^a^.

Districts, municipalities (map abbreviation)	Main village (n=9)	Distance to test (km)	Transit time (h/min)	District-wise geographic, demographic, and COVID-19 risk features
Phnom Srok (PS)	Nam Tau	75	2.5/150	Mountainous terrain Isolated No major roads Difficult and delayed access to testing, emergency, and specialty care High-risk area
Thma Puok (TP)	Kouk Romiet	65	2.17/130	Thai-Cambodia migration Border road crossing Immigration checkpoint Remote Distant from tertiary care Banteay Chhmar temple complex
Malai (M)	Tuol Pongro	60	2/120	Most villages near the border Border migration Checkpoint and crossing No major highways Difficult access Poor infrastructure
Krong Paoy Paet (new) (KPP)	Paoy Paet (Poipet)	53	1.77/100	Heavy bidirectional migrant worker and vehicle traffic across the border, which led to the spread of COVID-19 Poor access to testing High-risk casino area Densely populated In the process of redistricting
Svay Chek (SC)	Sla Kram	45	1.5/90	Border migration, checkpoints, and road crossings, which are difficult to control Rural areas with less-advanced transportation infrastructure Isolated from the main north-south road
Preah Netr Preah (PNP)	Phnum Lieb	40	1.33/80	Third most-populated district in the province Heavy transit to and from the border of Siem Reap Province, lead to increased carriers with SARS-CoV-2 Two major watercourses running through the district
Ou Chrov (OC)	Koub	31	1.03/62	On the main east-west conduit Border crossing Heavy foot and vehicle traffic with many feeder roads to the border Second-most populated Redistricting to balance resource use vs population density
Serie Saophoan (SS)	Kampong Svay	10	0.33/20	Numerous villages District with the fourth largest population (The municipality has a large population because of its central location and transport hub) Complex road grid Hospitals risking saturation Limited infection isolation facilities. Military rehabilitation center for young drug addicts
Monkol Borei (MB)	Banteay Neang	5	0.17/10	Most-populated district Location of COVID-19 sample collection site and province hospital Railroad transients to and from the border The average household size is 5.4 persons.

^a^Distance: median 45 (range 5-75) km, mean 42.7 (SD 23.9) km; transit time: median 90 (10-150) min, mean 84.7 (SD 47.6) min. Total province population: 853,252.

### Mobile-Testing Vans

Mobile and automated COVID-19 diagnostics in the rural Solano and Yolo counties of California presaged a framework of POC strategies and prehospital testing recommended for limited-resource settings in Cambodia. Coauthor AZ was the first to develop, implement, and manage COVID-19 testing in vans and transportable pop-ups starting in Vacaville (population 103,078), Solano County, in parallel with ongoing field research in Cambodia. Table S4 in Multimedia Appendix 1 summarizes test methods and operational features. Time to test result was 5 to 30 minutes. Patients received results immediately and through email. Vacaville was the site of the first community transmission of SARS-CoV-2 in the United States. Figure 4 in the study by Kost [32] presents the pictorial sequence of the first outbreak and the response from the Centers for Disease Control and Prevention. Mobile-testing vans in California were launched by CovidClinic, a nonprofit 501(c)(3) company that operated community testing at 78 locations in California and 140 clinics nationwide; in the discussion, we provide illustrations showing (1) the POCT design developed in the CovidClinic mobile laboratory and (2) the free-roaming-type clinic [42,43] operated by Optumserve. This roaming clinic provided COVID-19 onsite testing and prescription medications (Paxlovid and molnupiravir) via telehealth consultation for symptomatic patients who tested positive upon presentation at mobile clinic sites operated through February 2023 in Yolo County, which is adjacent to Solano County.

### Test Performance

In Figures S1 and S2 in Multimedia Appendix 1, we compare RAgT test performances. In 2021, RAgT developed by Roche Diagnostics met the WHO performance criterion for sensitivity >80% (median) in clinical evaluations (Figure S1 in Multimedia Appendix 1), while PanBio RAgT did not (Figure S2 in Multimedia Appendix 1).

## Discussion

### Principal Findings

This research identified a lack of sufficient COVID-19 diagnostics, both RAgTs and molecular diagnostics, in isolated and rural areas of Cambodia during peak outbreaks, excessive transit time to testing sites in border provinces and high-risk regions where international crossings are located, statistically significant differences in access to testing within border provinces, and geospatial gaps in access to testing in noncentral provincial areas. Implementation of novel diagnostic portals, such as mobile testing and 24-7 automated test dispensers in Yolo and Solano counties of California, provided models for public health delivery that would be feasible to implement in other rural settings including the limited-resource border regions studied in Cambodia. High performance POCT strategies allow timely, accessible, and mobile diagnosis of asymptomatic and symptomatic patients at critical points of need.

### Accessibility and Mobility of Diagnostics

COVID-19 demonstrated that weaknesses in health care small-world networks can generate adverse implications for vulnerable individuals, cultural welfare, workplace security, economic progress, and social instability [44-46]. Several thousand health care workers in the United States have died of COVID-19 [47]. The number of deaths in Cambodia is not known. Globally, the WHO estimated 115,500 COVID-19–related deaths among health care workers [48]. To protect medical professionals, first-time responders, oneself, families, and others from acute and protracted disease, especially to protect individuals older than 65 years (among whom 90% of COVID-19–related deaths were reported in November 2022 [49]), accessible COVID-19 diagnostics and rapid-test results remain paramount.

The COVID-19 pandemic inspired several mobile-testing solutions. In China, Xing et al [50] developed a mobile laboratory for onsite molecular diagnostics. Others deployed inflatable molecular diagnostic laboratories in Beijing [51]. In Singapore, Linster et al [52] published a mobile design and highlighted its biocontainment features. In Victoria, Australia, Ballard et al [53] described a testing van with ISO 15189 certification and illustrated testing process steps (see Figure 1 in the study by Ballard et al [53]). In addition, in Australia, Paton et al [54], with the assistance of Lab Without Walls, deployed mobile molecular diagnostics for the detection of SARS-CoV-2.

In Angola, Africa, Owens et al [55] devised a mobile laboratory curriculum. In the United States, Kost et al [56] created a comprehensive POCT educational curriculum for public health learners. In South Korea, Roh et al [57] compiled guidelines for mobile testing comprising installation, equipment, evaluation, sample processing or storage, testing or reporting, internal or external quality control, management, personnel, infection control, biosafety, and information systems. Affara et al [58] described mobile laboratory networks in Africa. In East African countries, Gehre et al [59] showed that mobile testing providing certifications of uninfected drivers helped facilitate business enterprise when borders closed. Tong et al [60] used spatial modeling to help control COVID-19 in Hong Kong. However, extensive literature searches did not recover any reports of COVID-19 mobile-testing laboratories or geospatial analysis of testing sites in Cambodia.

**Figure 5:**
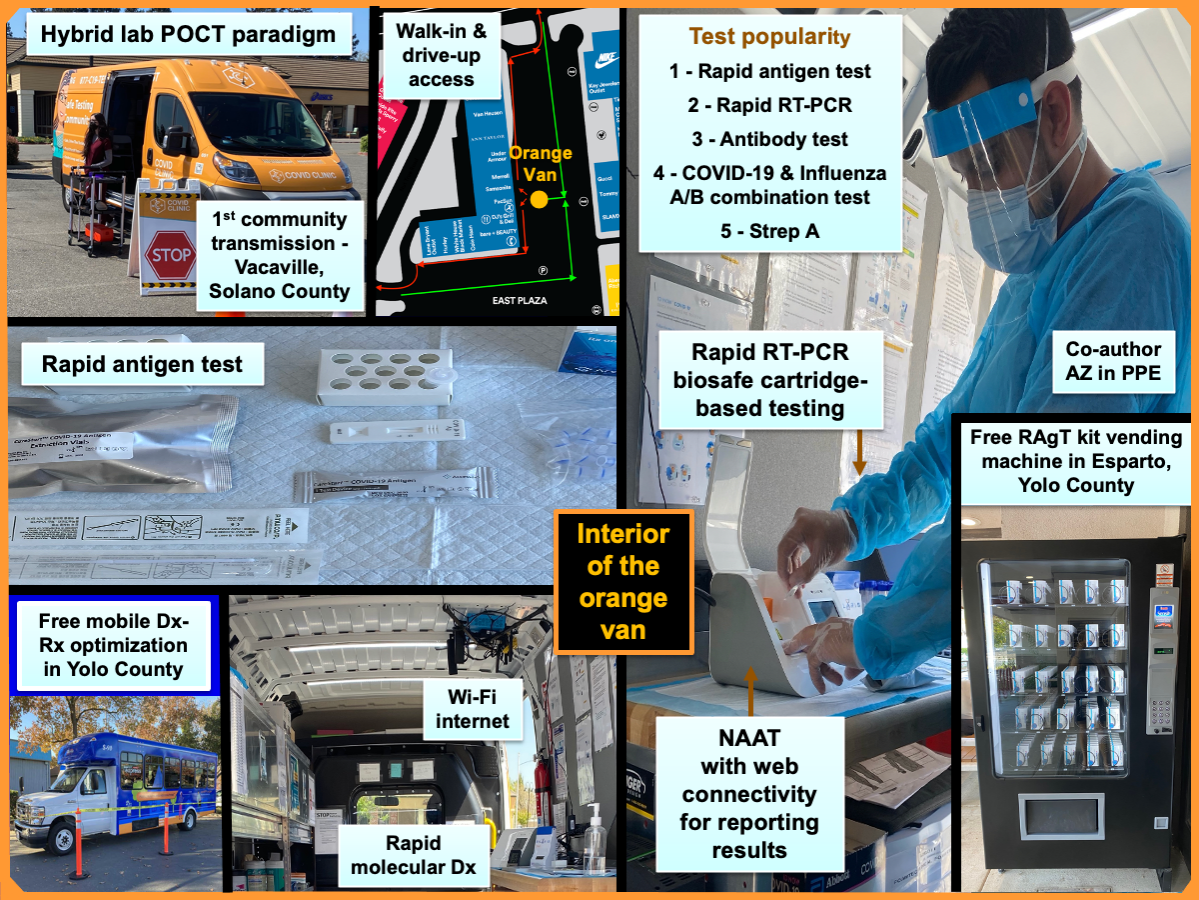
Mobile laboratory-POC strategies with telehealth implemented during the pandemic in rural Solano and Yolo counties of Northern California. The frames show the walk-up van, test popularity, and assay bench. For biosafety, molecular testing is cartridge based. The bottom right displays one of the several RAgT vending machines that anonymously issue free-of-cost test kits in Yolo County. The bottom left shows a mobile clinic for diagnostic-therapeutic (Dx-Rx) optimization, that is, COVID-19 diagnosis with Paxlovid or Molnupiravir treatment to care for patients with positive COVID-19 test results. Dx-Rx: diagnostic-therapeutic; NAAT: nucleic acid amplification test; POCT: point-of-care testing; PPE: personal protective equipment; RAgT: rapid antigen test.

Mobile-testing vans provide diagnostic portals [10], multiplex testing, professional personnel, quality assurance, and telehealth. Accessible diagnostics (Figure 5) in Solano and Yolo counties in California fulfilled public health needs for distributed COVID-19 testing and surveillance in these rural regions. From July 2022 to February 2023, a total of 51,000 RAgT kits were given out [61]. The mobile clinic with telehealth consultation started operating in Yolo County during the fall surge of 2022 as one element of the California “Test-to-Treat” program. Free masks (ie, N95) can be added to these accessible resources, as well as other public health supplies, such as mosquito repellents, sunscreens, condoms, pregnancy tests, sexually transmitted infection tests, and overdose reversal drugs such as naloxone [61]. Personal protective equipment must be worn in mobile-testing zones, which are considered active laboratories, vans must maintain appropriate ambient environmental temperature and biosafety measures, and in the United States the Occupational Safety and Health Administration requires fire escape plans.

### Meeting Challenges

Of Cambodia’s 17.2 million people, approximately 80% live in rural areas in which mobile services can improve health care. Transit time, return trips, and lost wages diminish motivation to make arduous trips to referral hospitals for testing [62]. Rural communities have a higher proportion of older adults than urban communities; the frequency of noncommunicable diseases is higher among the rural poor [63-66]. Migrant workers crossing borders spread COVID-19. Sparsely placed rural health services and infrastructure made it difficult to effectively respond to COVID-19, its variants, and confounding diseases [67], which, if neglected, can result in excess mortality.

Table 3 summarizes challenges encountered when implementing mobile van testing and lists recommendations for implementation of POCT in rural and limited-resource settings. In early May 2021, during the height of the COVID-19 surge in Cambodia, we recommended [62] free distribution of RAgTs for self-testing throughout villages and mobile RAgTs and RT-PCR testing at key locations (indicated by the red dots in Figure 3) including border check points, to identify infections where people crossed borders to reach markets, work, and casinos. Generally, one-way distances and travel times from Banteay Meanchey villages to RT-PCR testing were too prolonged (median 45 km; Figure 4) to motivate travel to molecular diagnostic testing sites.

**Table 3:** Mobile-testing benefits with recommendations that assure high-quality diagnostic performance for prehospital and POC^a^ diagnosis in limited-resource settings^b^.

Challenges		Rationale and recommendations
**Strategic planning**		
	Test menus that meet public health needs	A mobile diagnostic portal does not need to focus strictly on providing COVID-19 testing across geographic regions, although the number of tests offered in mobile laboratories is limited due to space constraints. The inclusion of wellness testing, such as tests for HbA_1c_^c^, TB^d^, HIV, gonorrhea, HPV^e^, and other STDs^f^, helps serve broad community needs before, during, and after outbreak responses.
	Regional and local priorities	To the extent possible, mobile resources can be integrated into regional and local strategic plans for testing. Geospatial analyses and transit time metrics help optimize resource positioning and geospatial care paths.
**Community outreach**		
	Education and accurate information	At the height of the COVID-19 pandemic, misinformation was abundant. Mobile clinics can teach health care providers, educate patients, and help interpret test results to compensate for the disproportionate ratio of patients to medical personnel in the community.
	Risk avoidance vs risk management	Mobile testing helps transition risk avoidance to management by rapidly identifying contagion, determining the geospatial distribution of outbreaks, and limiting dissemination associated with human migration. Telehealth and licensed providers can accelerate treatment for symptomatic, diagnosed patients. Local vaccination reduces risk by limiting the spread of the disease in border, rural, and urban areas.
**Testing process**		
	Timeliness and connectivity	Technological issues that delay patient registration via the web or delivery of results to patients may occur often. Network connectivity will improve the timeliness of the result reports.
	Reagent storage	Reagents and test kits should be stored within manufacturer-specified temperatures and humidity specifications once service hours end. Suitable ambient conditions, including along reagent supply chains, should be carefully monitored and maintained 24-7.
	Reference laboratory testing	Minimal out-of-pocket options, such as laboratory-based RT-PCR^g^ funded by United States, international, or local health care plans, offer affordable diagnosis for patients. However, the referral laboratory turnaround time may be 2-14 days, which increases the risk of contagion. Consider sending patients to local mobile sites offering equivalent economical test options.
**Health geographics**		
	Geospatial mapping and site selection	Geospatial mapping helps optimize resource locations with high human traffic. Mobile clinics create high value in remote areas. However, patients who require urgent care may not locate mobile sites. In high traffic areas mobile clinics should stand out from other vehicles and be safely accessed by foot, motorcycles, and other vehicles.
	Physical constraints	Mobile clinic interior compartments limit space available for storing tests and the number of instruments on the testing bench. Therefore, select vehicles with ample space inside.
**Environment concerns**		
	Temperature and humidity fluctuations	Seasonal weather may lead to temperature fluctuations and humidity levels that exceed manufacturer specifications. Excessively low and high temperatures in testing areas should be counteracted with heating and cooling to perform tests, provide quality control, and validate results properly.
**Logistics**		
	Registration	Scannable paper-based phone apps and desktop interfaces required for patients to share pertinent medical history, symptoms, and contact information need to be adapted for community populations, languages spoken, and different cultural contexts.
	Insurance and billing	Declaration of COVID-19 as a public health emergency facilitates the coverage of substantial zero-cost RT-PCR testing. Some POC tests are not covered unless they are performed within primary care offices. Funding of mobile resources must be coordinated at regional levels.
	Signage	Signage should be protected from wind and inclement weather, especially if the mobile clinic does not have a distinct physical address.
**Technical support**		
	Electric power	Operations rely on portable battery packs, fuel supplies, and solar panels to operate instruments and lighting. These energy resources require medical operators to set up, renew, and charge at the end of the day. Therefore, there must be appropriate training protocols.
	Networking, Wi-Fi, and connectivity	A mobile hotspot (via phone) or portable Wi-Fi Datto box can transmit test results and receive patient documents from base stations. Technological issues can delay and prevent testing from occurring. Therefore, plan alternate sites and alert patients.
**Outcomes**		
	Public health	Diagnostic test results generated by mobile vans and vending machines should be linked with outcomes and documented to enhance understanding for future implementations. For example, the United States National Institutes of Biomedical Imaging and Bioengineering (NIH^h^ Radx Tech Initiative) offered to “Make My Test Count,” a website for anonymous reporting of self-testing results. For high community impact, we recommend that local public health agencies document and report testing and outcomes data daily during outbreaks and surges.
	National guidelines and policy	National policy and guidelines can support mobile and distributed diagnostics for highly infectious threats. On the basis of the knowledge learned during the COVID-19 pandemic, consensus guidelines and policy can be developed to prepare for future public health crises.

^a^POC: point-of-care.

^b^US mobile laboratories are inspected by the Centers for Medicare and Medicaid Services and the Occupational Safety and Health Administration. In California, mobile laboratories are Clinical Laboratory Improvement Act waived. A physician at CovidClinic signed test results before their release to patients. Mobile vans operate with local business licenses. Business licenses and Clinical Laboratory Improvement Act documents must be posted.

^c^HbA_1c_: hemoglobin A_1c_.

^d^TB: tuberculosis.

^e^HPV: human papillomavirus.

^f^STD: sexually transmitted disease.

^g^RT-PCR: reverse transcriptase polymerase chain reaction.

^h^NIH: National Institutes of Health.

### Fulfilling Needs and Building Public Health Resilience

Awareness of infection through COVID-19 testing can help meet several identified needs. Transmission could have been slowed by checkpoint and self-testing to detect COVID-19 variants such as Delta in 2021, and later Omicron, which spread quickly across Thai-Cambodia borders. Unfortunately, the costs (US $2-$7) of most RAgTs were prohibitive for rural Cambodians at the time of 2021 peak surges. Test kits were not generally available nor distributed freely in rural areas. Eventually, the Cambodian government approved and distributed limited supplies of RAgTs, which we encouraged through communications, lectures, and webinars with the National Public Health Laboratory staff, university, and professional participants.

Rapid self-testing and timely awareness can help people adapt to endemic diseases that demand long-term medical and social change [68]. Empowered with immediate test results, people can receive guided treatment using telehealth to optimize diagnostic-therapeutic cycles [69]. Geospatial strategies in limited-resource settings should address “spatial justice,” that is, “the fair and equitable distribution in space of socially valued resources and the opportunities to use them” [70]. Home, community, and emergency testing [10,40], including layperson self-testing and testing among families at an affordable cost or subsidized cost by government agencies can help establish spatial justice for rapid diagnosis. Benefits of bedside, mobile, door-to-door, and prehospital testing comprise high-volume testing; improved access; privacy; multiple test formats; service for hard-to-reach, marginalized, vulnerable, and underserved people; diminished disparities and inequities in mortality rates; tracking of test positivity rates; outbreak mitigation; fulfilling needs in isolated island and regional rural settings [71-81]; and improving patient outcomes [82-84].

For high-risk border provinces and rural outbreaks, disparities in access justify distributing and mobilizing testing. Even now, quick access to COVID-19 and other infectious disease tests could help avoid older adult deaths and the spread of pediatric infections. A sustainable business model might be enabled by including tests for infectious disease surges, such as seasonal influenza A and B (2022, highest cases in over a decade), respiratory syncytial virus with portable multiplex molecular assays, and increasingly prevalent community infections such as strep throat [85]. In rural communities threatened by de novo pathogen transmission, multipurpose mobile testing, automated test kit access, low-cost diagnostics, data-driven surveillance systems [86], and telehealth treatment today seem a rational way to prepare for tomorrow. Crisis standards of care [87,88] would help prepare, codify, sustain, and enhance the use of novel POC strategies in anticipation of the next pandemic.

### Limitations

This research successfully documented geospatial constraints in access to COVID-19 testing in rural Cambodian border provinces. Early POC diagnosis helps find new pathogens, limits their spread, and informs public health of increasing threats [89]. However, limited scope and government restrictions prevented the discovery of how outcomes were affected by changes in spatial testing patterns during and following COVID-19 surges in 2021. The Cambodian government approved the use of RAgTs in private hospitals and clinics in late April 2021 [90] and, on July 7, 2021, approved RAgT self-testing in homes with Roche, PanBio, Standard Q, and Indicaid kits [91]. Although the Ministry of Health published a RAgT operating procedure for border gates, factories, and other business locations in July 2021 [92], the testing rate in Cambodia was too slow to keep pace with the spread of new viral variants (eg, Delta in 2021 and later, Omicron).

Then, in February 2022, the government declared it a crime if citizens withheld positive COVID-19 test results [93]. On October 1, 2021, the government stopped administering RAgTs and ceased reporting RAgT-positives; case numbers reported fell 76% [94]. Consequently, by March 2023, Cambodia ranked 125th worldwide for COVID-19 cases (138,719) with 3056 reported deaths (104th), 94th for total tests (3,091,420), but 162nd for tests per 1 million population (180,062/million) [11]. Notwithstanding government policies, RAgTs cannot reliably rule out COVID-19. The US Food and Drug Administration has approved home tests for simultaneous detection of COVID-19 and influenza [95]. However, home molecular diagnostics (eg, loop‐mediated isothermal amplification), which demonstrates higher levels of performance for ruling out and ruling in infectious diseases, should be taught in public health curricula [96,97] and set expectations for future home testing [98,99]. To mitigate these limitations, we also recommend that national guidelines for high performance and cost-effective diagnostics [100] be put in place proactively in limited-resource settings to anticipate emerging variants, new outbreaks, and a possible future pandemic, as well as to improve the ongoing accessibility of COVID-19 and other vital diagnostics for highly vulnerable populations, such as people living with HIV [101].

### Conclusions and Recommendations

The COVID-19 crisis propelled POCT directly into communities to meet the needs of people for quick access to testing in homes and at nearby diagnostic portals, although as noted earlier, local prevalence and the presence of symptoms influence RAgT reliability. Experience with rural American mobile testing and automated test kit dispensing suggests that these approaches could be implemented in limited-resource rural provinces in Cambodia, in remote settings of other countries, and in island nations to build public health resilience. Hence, public health practitioners should be prepared to implement POCT strategically in communities and homes, enable prehospital mobile testing, provide automated test dispensers, establish telehealth consultation, and expedite treatment when indicated by positive diagnostic results obtained rapidly at points of need.

Automated vending machines that dispense COVID-19 RAgTs and home molecular diagnostics kits (eg, loop‐mediated isothermal amplification tests) 24-7 could be positioned in public health vehicles and POCT vans as shown in Figure 5, facilitating safe testing and reliable detection of SARS-CoV-2 or new emerging viruses and variants in hotspots in which symptoms are experienced first. Targeted vaccination, follow-up community testing, travel certificates, telecommunicated epidemiological warnings, and the addition of public health data to surveillance repositories represent additional potential benefits. Mobility could be extended to wellness testing to cost-effectively enhance public health outreach in underserved areas.

Quick detection, control, and mitigation of outbreaks require adaptable, flexible, and dynamic responses that fit geographic, social, environmental, seasonal, and cultural expectations. When not responding to infectious diseases or other crises, mobile and prehospital POCT could enable rapid response care close to homes in rural and remote areas while playing an important role in educating health care providers about POCT field practice and its advantages. Public health facilities, budgets, and capabilities differ from country to country. Therefore, national POCT guidelines should be written in anticipation of infectious disease crises to accelerate regional responses and their geospatial optimization.

RAgTs are more effective when symptoms and viral load peak. Testing within communities at optimal sites determined by quantitative geospatial analysis would help close gaps in public health accessibility, reveal health care discrepancies, and speed diagnostic test results in limited-resource areas. Geospatial planning can enhance resilience, assure spatial justice, and deliver diagnostic testing to highly vulnerable populations such as people living with HIV. Public health assets should be characterized by higher-performance, lower-cost, readily accessible, and user-friendly POC technologies such as molecular diagnostics for self-testing and distributed border detection for public health surveillance.

## Appendix

Multimedia Appendix 1 Supplementary digital content.

## Abbreviations

POCT: point-of-care testing
RAgT: rapid antigen test
RT-PCR: reverse transcriptase polymerase chain reaction
WHO: World Health Organization

## Data Availability

The data sets generated and analyzed during this study are available from Knowledge Optimization on reasonable request.
