# Supplementary material for: Geospatial Point-of-Care Testing Strategies for COVID-19 Resilience in Resource-Poor Settings: Rural Cambodia Field Study
Source: JMIR Public Health Surveill. 2024 Aug 27;10:e47416. doi: 10.2196/47416 (PMC11387922; doi:10.2196/47416)
Supplement: Multimedia Appendix 1 [file publichealth_v10i1e47416_app1.pdf]

SUPPLEMENTAL DIGITAL CONTENT

GEOSPATIAL POINT-OF-CARE TESTING STRATEGIES FOR COVID-19 RESILIENCE IN RESOURCE-POOR SETTINGS: RURAL CAMBODIA FIELD STUDY

Table of Contents **Page**

Focus Group Questionnaire [English]……………………………………… 1
Focus Group Questionnaire [Khmer]………………………………………. 5
Rapid Antigen Test Performance Evaluations [2021]……………………… 12
Theory, COVID-19 Rapid Antigen Tests in Cambodia……………… 12
Figure S1. Roche Diagnostics Rapid Antigen Test Performance……... 12
Table S1. Roche Rapid Antigen Test Clinical Evaluations…………… 13
Figure S2. PanBio Rapid Antigen Test Performance………………….. 14
Table S2. PanBio Rapid Antigen Test Clinical Evaluations…………... 15
Conclusions Regarding COVID-19 Test Performance………………... 17
Table S3. Mathematical Equations.…………………………………………. 18
Table S4. COVID-19 Tests Performed in the Mobile Hybrid Laboratory-POCT Van for Rural Communities during the Pandemic in Northern California……………………………………………………………… 20
References — The Mathematics of COVID-19 Diagnostic Tests……………... 21

FOCUS GROUP QUESTIONNAIRE [ENGLISH]

EIGHT FOCUS GROUP TOPICS FOR FEEDBACK ON COVID-19 POINT-OF-CARE (POC) STRATEGIES

Focus Topic 1—Cambodian Provinces in Highest Danger. Please identify the top five provinces or regions (e.g., international borders) in Cambodia (outside of Phnom Penh) with the highest risk of collapse of the local or regional healthcare systems from continuing and worsening COVID-19 contagion and overloading of local healthcare resources.

1.______________________________ (highest risk)
2.______________________________
3.______________________________
4.______________________________
5.______________________________ (less risk)

Focus Topic 2—COVID-19 Diagnostic Testing Strategies. For the top 3 provinces listed above, please state what you think are the most effective COVID-19 diagnostic testing strategies in order of priority (1, highly effective; 5, not very effective).

Strategy 1.____

Strategy 2.____

Strategy 3.____

Strategy 4.____

Strategy 5.____

Are there other strategies that you recommend? __ Yes ___ No

If "yes," please explain what they are:______________________________________

Focus Topic 3—The Role of COVID-19 Rapid Antigen Tests. Diagnostic results from rapid COVID-19 antigen tests are available in a few minutes. Please describe a) the purpose of these tests in provinces with the highest prevalence of COVID-19 infections, b) the provinces where rapid antigen testing has been implemented already, c) the types of geographical sites within those provinces where the tests are located, and d) the manufacturers of the antigen tests.

---

a) Purpose of antigen testing—

b) Where used, such as immigration, emergency rooms, clinical laboratory, or other—

c) Types of geographic sites—

d) Manufacturer/name of tests (e.g., Roche Rapid Antigen Test, Abbott Panbio, others)—
i)______________________________ii)______________________________
iii)______________________________v)______________________________

Focus Topic 4—Placement of Testing. Each province is characterized by its "small-world network," that is, the type of healthcare system in the context of the local culture, communications among people and professionals, the road network, the emergency medical system, ambulance routes, locations of emergency rooms, hospital capacity, availability of ICU beds (if any), and other key factors, such as mountainous geography.

Where in rural healthcare small-world networks, do you recommend COVID-19 testing — molecular diagnostic ("PCR"), rapid antigen ("Ag"), or antibody ("Ab") testing — should be

placed? Please be specific and include a map of the highest risk province you have identified earlier with testing locations marked on the map of the province.

Identify the map you marked up:____

Mark your placement of testing types as: "PCR," "Ag," or "Ab."

If you do not believe rapid or local testing would help, please describe your system for obtaining COVID-19 swab specimens, where you would send them for evaluation, and how long it would take:

____
____
____

Focus Topic 5—Mobile COVID-19 Testing. Please describe when, where, and how mobile POC testing in a van or other vehicle or mobile health unit could be used in Cambodia. Identify provinces, actual testing sites, on-site sample collection, personnel needed, if you have participated personally, when you did that, and the future of mobile COVID-19 testing in rural areas.

Description:____
____
____
____
____
____

Focus Topic 6—Selection of Instruments and Test Clusters for Mobile COVID-19 Testing. Below is a picture of the instrument bench and testing area inside a mobile van used in California USA. Please list the instruments (or disposal kits) and the tests you would place inside a Cambodian mobile unit for RURAL regions, in order of priority—

	Instrument or Lateral Flow Assay	Diagnostic Test(s) It Performs
1st Choice		
2nd Choice		
3rd Choice		
4th Choice		

Photo of the inside of the California van—

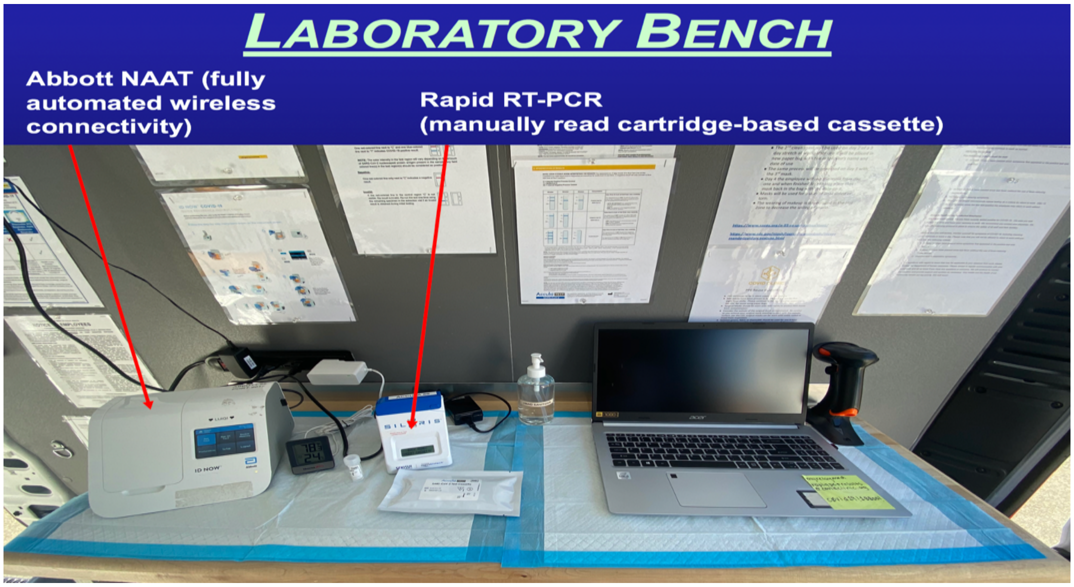

Focus Topic 7—Instrumentation and Future Designs. We should support health providers when they select COVID-19 diagnostics for different settings, environmental conditions, and community prevalence. Testing should be fast, so results are known quickly for quarantine, contact tracing, and possibly, hospitalization.
Please describe your design of the IDEAL COVID-19 TESTING DEVICE. Be creative! Draw a picture! State the device specifications and the assays the device will perform.

The specifications for my design, in order of importance ("a" most important), are:
a)____
b)____
c)____
d)____
e)____
The assays it will perform are:
1)____________________________2)_______________________________
3)____________________________4)_______________________________

Here is a design sketch of my ideal instrument! Label the dimensions and size; whether handheld, portable, or transportable; and the cost to purchase and operate. Design sketch—

Focus Topic 8—National POC Testing Policy and Guidelines ("P&G"). Do national COVID-19 point-of-care testing policy and guidelines have a role in Cambodia? Is anyone writing such a document for Cambodia? Let's assume we are drafting the document. Please construct the following:

Role of the P & G document in the COVID-19 crisis:____________________________________

____

Goals for national POC testing P & G in Cambodia:

1. ____
   ____
2. ____
   ____
3. ____
   ____

Is such a document in preparation? ___ Yes ___ No. If yes, who is preparing it? ____________

____

FOCUS GROUP QUESTIONNAIRE [KHMER]

8 សំនួរដើម្បីប្រមូលមតិយោបល់ទាក់ទងទៅនឹងយុទ្ធសាស្ត្រនៃការថែទាំជិតអ្នកជំងឺ កូវីដ-19

FEEDBACK ON COVID-19 POINT-OF-CARE (POC) STRATEGIES

ប្រធានបទទី1 : ខេត្តនៃប្រទេសកម្ពុជា ដែលមានហានិភ័យខ្ពស់ដោយជំងឺកូវីដ-19។ បើសិនជា ជំងឺកូវីដ-19នៅតែបន្ត ហើយការឆ្លងកាន់តែរីករាយដាលខ្លាំងទៅៗ នោះប្រព័ន្ធសុខាភិបាលជាពិសេសនៅតាមខេត្តប្រឈមមុខទៅនឹង ការពិបាកគ្រប់គ្រងដោយសារធនធានមានកំណត់។ ក្នុងករណីនេះ ចូរអ្នកបង្ហាញខេត្តនៃប្រទេសកម្ពុជា 5 ក្នុងចំណោម 25 ខេត្ត-ក្រុង ដែលមានមានហានិភ័យខ្ពស់ជាងគេ ពីលំដាប់1 ហានិភ័យខ្ពស់បំផុត ទៅលំដាប់5 ហានិភ័យខ្ពស់តិចតួច។

ចម្លើយ:

1) .... **(ហានិភ័យខ្ពស់បំផុត)**
2) ....

3) ....
4) ....
5) .... **(ហានិភ័យខ្ពស់តិចតួច)**

ប្រធានបទទី2: យុទ្ធសាស្ត្រនៃការធ្វើរោគវិនិច្ឆ័យជំងឺកូវីដ-19។ ចូរចំណាត់ថ្នាក់យុទ្ធ-សាស្ត្រនៃការធ្វើរោគវិនិច្ឆ័យជំងឺកូវីដ-19 ចំនួន5 ដែលអ្នកគិតថា មានប្រសិទ្ធភាពខ្ពស់ជាងគេ និងអាចអនុវត្តបាននៅក្នុងខេត្ត3ខាងលើដែលមានហានិភ័យខ្ពស់ខ្លាំង។

ចម្លើយ:

យុទ្ធសាស្ត្រទី1:...
....

យុទ្ធសាស្ត្រទី2:
....
....

យុទ្ធសាស្ត្រទី3:
....
....

យុទ្ធសាស្ត្រទី4:
....
....

យុទ្ធសាស្ត្រទី5:
....
....

តើមានយុទ្ធសាស្ត្រផ្សេងទៀតដែលអ្នកណែនាំទេ? __ មាន __ អត់ទេ

បើ“ មាន” សូមពន្យល់ថាមានអ្វីខ្លះ៖
....
....

ប្រធានបទទី3: តួនាទីនៃការធ្វើតេស្តអង់ទីហ្សែនរហ័សនៃ ជំងឺកូវីដ-19។

ការធ្វើរោគវិនិច្ឆ័យតាមរយៈការធ្វើ តេស្តអង់ទីហ្សែនរហ័ស នៃជំងឺកូវីដ-19 អាចផ្តល់លទ្ធផលក្នុងរយៈពេលពីរទៅបីនាទីតែប៉ុណ្ណោះ។ ចូរពិពណ៌នា:

1) គោលបំណងនៃការធ្វើតេស្តទាំងនេះនៅតាមបណ្តាខេត្តដែលមានអត្រាឆ្លងរាល-ដាលខ្ពស់បំផុតនៃជំងឺកូវីដ-19។

2) ផ្តល់ឈ្មោះខេត្ត និងទីតាំងជាក់លាក់ដែលមានការអនុវត្តន៏ធ្វើតេស្តអង់ទីហ្សែន រហ័សរួចហើយ។

3) ប្រភេទនៃទីតាំងភូមិសាស្ត្រនៅក្នុងខេត្តទាំងនោះដែលមានកន្លែងធ្វើតេស្តទាំង នេះ។

4) ផ្តល់ឈ្មោះក្រុមហ៊ុនផលិតតេស្តអង់ទីហ្សែនរហ័សដែលប្រើប្រាស់ក្នុងប្រទេសកម្ពុជា។

ចម្លើយ:

1) គោលបំណងនៃការធ្វើតេស្តអងទីហ្សែនរហ័ស:

....

....

2) ឈ្មោះខេត្ត និងទីតាំងដាក់លាក់ដែលផ្តល់ការធ្វើតេស្តអង់ទីហ្សែនហ័ស:

....

....

3) ប្រភេទនៃទីតាំងភូមិសាស្ត្រនៅក្នុងខេត្តទាំងនោះដែលមានកន្លែងធ្វើតេស្ត:

....

....

4) ផ្តល់ឈ្មោះក្រុមហ៊ុនផលិតតេស្តអង់ទីហ្សែនទាំងនោះ:

....

....

ប្រធានបទទី4: ទីតាំងនៃការធ្វើតេស្ត។ ខេត្តនីមួយៗត្រូវបានកំណត់លក្ខណៈជាបណ្តាញពិភពលោកតូចមួយ។ បណ្តាញពិភពលោកតូច ជាប្រភេទនៃប្រព័ន្ធថែទាំសុខភាពក្នុងបរិបទវប្បធម៌ក្នុងស្រុកនៃតំបន់នោះ ដែលមានការប្រាស្រ័យទាក់ទងរវាងប្រជាជននិងគ្រូពេទ្យ ផ្លូវគមនាគមន៍ មានរថយន្តសង្គ្រោះបន្ទាន់ ទីតាំងសង្គ្រោះបន្ទាន់ គ្រែអាយស៊ីយូ (បើមាន) និងកត្តាសំខាន់ៗផ្សេងទៀត ដូចជាភូមិសាស្ត្រភ្នំ ឬព្រៃដែន។ តើកន្លែងដាក់លាក់ណាមួយនៃបណ្តាញពិភពលោកតូចនេះ នៃខេត្តមួយដែលអ្នកគិតថាមានហានិភ័យជំងឺកូវីដ-19 ខ្ពស់បំផុត (យោងតាមសំនួរនៃប្រធានបទទី1) ដែលអ្នកណែនាំអោយមានការធ្វើតេស្តកូវីដ-19 ដែលរួមមានការធ្វើរោគវិនិច្ឆ័យម៉ូលេគុល (PCR), តេស្តអង់ទីហ្សែនរហ័ស (Ag) ឬតេស្តអង់ទីកររហ័ស (Ab)? ចូរសាកល្បងដាក់ទីតាំងដាក់លាក់នៃតេស្តនីមួយៗ នៅលើផែនទីខេត្តដែលមានហានិភ័យខ្ពស់បំផុតនោះ។ ជាជំនួយ អ្នកអាចទាញយកផែនទីនៃខេត្តប្រទេសកម្ពុជាតាមរយះ (https://hellocambodia.wordpress.com/about/maps/road-maps/)។ បន្ទាប់មក អ្នកអាច Copy ផែនទីមួយក្នុងចំនោមផែនទីទាំងនេះ ដាក់ចូលក្នុងក្នុងចម្លើយ រួចអ្នកព្យាយាមដាក់គំនូសតាងលើផែនទី “PCR” “Ag” or “Ab” នៅលើទីតាំងដែលអ្នកគិតថាគួរតែមានការផ្តល់នៅតេស្តម៉ូលេគុល តេស្តអង់ទីហ្សែនរហ័ស និងតេស្តអង់ទីកររហ័ស ទៅតាមទីតាំងដាក់លាក់ដែលអ្នកគិតថាវាជាទីតាំងល្អដែលអនុញ្ញាតិអោយប្រជាជនក្នុងតំបន់នោះ អាចទទួលបានភាពងាយស្រួលក្នុងការធ្វើតេស្តទាំងនេះ។ ចំណាំ អ្នកអាចដាក់តេស្តរហ័សទាំងនេះ

នៅច្រើនកន្លែងបាន បើសិនជាអ្នកគិតថា វាអាចបង្កភាពងាយស្រួលដល់ប្រជាជនក្នុងខេត្តនោះ ទាំងក្នុងក្រុង និងតំបន់ជនបទ។

ចម្លើយ:

ផែនទី ទីតាំងការធ្វើតេស្ត:

....
....
....
....
....
....
....
....
....

ក្នុងករណីប្រសិនបើ អ្នកមិនគិតថាការធ្វើតេស្តិ៍ក្នុងតំបន់ អាចជួយពន្លឿនលទ្ធផលបានទេនោះ ចូរអ្នកពណ៌នាប្រព័ន្ធមួយដែលអ្នកគិតថាអាចទទួលបានលទ្ធផលលឿន តាមរយៈការធ្វើ សំណាក swab ទៅវិភាគនៅកន្លែងដែលអ្នកគិតថានឹងទទួលបានលទ្ធផលរហ័ស ដោយបញ្ជាក់ពីពេលវេលាក្នុងការផ្តល់លទ្ធផលផងដែរ។

....
....

ប្រធានបទទី5: **ការធ្វើតេស្តកូវីដ-19ចល័ត ជាការធ្វើតេស្តនៅលើរថយន្ត ដែលអាច បើកបរចល័តបាន ដើម្បីផ្តល់ការធ្វើតេស្តដល់ប្រជាជន។** តើអ្នកគិតថា ម៉ូដែលនៃការធ្វើតេស្តចល័តបែបនេះ អាចអនុវត្តបាននៅក្នុងប្រទេសកម្ពុជាបានដែរឬទេ? បើបាន តើគួរតែអនុវត្តនៅកន្លែងណា ឬតំបន់ណា ខេត្តណា? ហើយតើក្នុងរថយន្តចល័តនោះ គួរដាក់នូវតេស្តកូវីដ-19ប្រភេទណាខ្លះ?

ចម្លើយ:

..
..
....

ប្រធានបទទី6: ការជ្រើសរើសឧបករណ៍ និងក្រុមតេស្តសំរាប់ការធ្វើតេស្តិ៍កូវីដ-19ចល័ត។
ខាងក្រោមនេះគឺជារូបភាពនៃឧបករណ៍ និងក្រុមតេស្តនៅខាងក្នុងឡានចល័តដែលត្រូវបានប្រើនៅរដ្ឋកាលីហ្វ័រញ៉ានៃប្រទេសសហរដ្ឋអាមេរិក។ ចូរអ្នករាយឈ្មោះឧបករណ៍ និងប្រភេទតេស្តដែលអ្នកនឹងដាក់នៅយានយន្ត សម្រាប់ការធ្វើតេ-ស្តិ៍កូវីដ-

19នៅតាមតំបន់អនុភាពនៃប្រទេសកម្ពុជា។

ចម្លើយ:

ជម្រើស	ឧបករណ៍	ក្រុមតេស្ត
1)		
2)		
3)		
4)		

នេះជារូបភាពនៃឧបករណ៍

និងក្រុមតេស្តនៅខាងក្នុងឡានចល័តដែលត្រូវបានប្រើនៅរដ្ឋកាលីហ្វ័រញ៉ានៃប្រទេសសហរដ្ឋអាមេរិក :

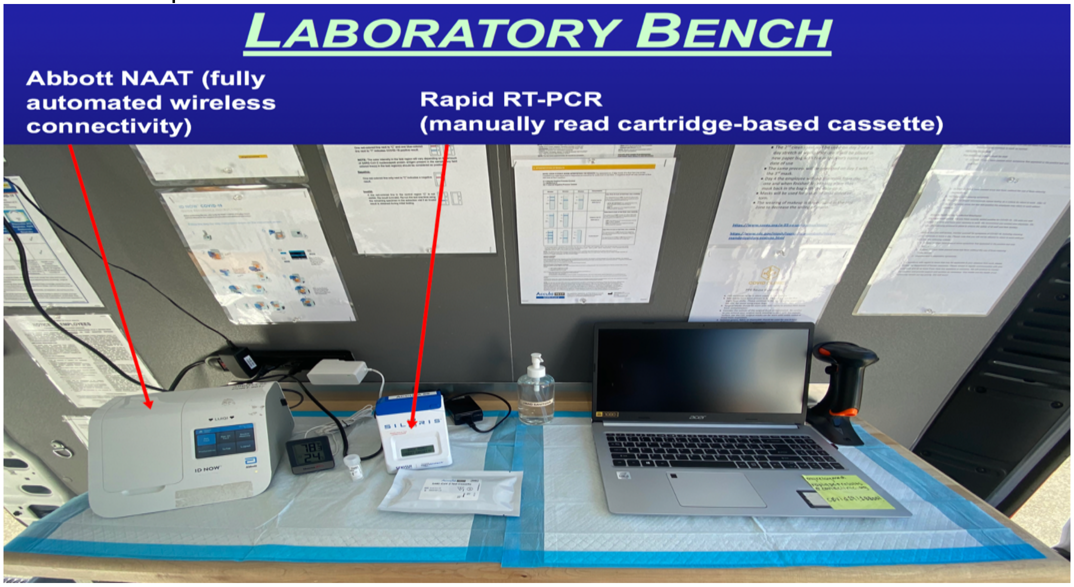

ប្រធានបទទី7 : ឧបករណ៍សម្រាប់ការធ្វើតេស្តបច្ចុប្បន្ន និងការរចនាឧបករណ៍សម្រាប់ការធ្វើតេស្តនាពេលអនាគត។ យើងគួរតែគាំទ្រអ្នកផ្តល់សេវាសុខភាព ដោយផ្តល់នូវគោលការណ៍អនុវត្តន៍ត្រឹមត្រូវ ក្នុងការជ្រើសរើសការធ្វើរោគវិនិច្ឆ័យជំងឺកូវីដ-19 ទៅតាមបរិបទនៃការប្រើប្រាស់ផ្សេងៗគ្នា ក្នុងលក្ខខណ្ឌបរិស្ថាន និងអត្រាប្រេវ៉ា-ឡង់សហគមន៍ខុសៗគ្នាជាដើម។ ការធ្វើតេស្តគួរតែមានរយះពេលលឿន ដូច្នេះលទ្ធផលដែលបាន អាចអោយគេដឹងបានឆាប់ ថាតើអ្នកធ្វើតេស្តនោះគួរតែ សម្រាកដាច់ដោយឡែកពីគេ រក្សាគម្លាតទំនាក់ទំនង ឬត្រូវស្នាក់នៅមន្ទីរពេទ្យ។ ដោយប្រើប្រាស់គំនិតច្នៃប្រឌិតរបស់អ្នក

ចូរអ្នកពណ៌នាការរចនាផ្ទាល់របស់អ្នកនូវឧបករណ៍ធ្វើតេស្តកូវីដ-19។ បើអាច ចូរគូររូប ព្រមទាំងបញ្ជាក់ពីលក្ខណៈបច្ចេកទេសរបស់ឧបករណ៍ និងតេស្តដែលប្រើដើម្បីដំណើរការ។

ចម្លើយ:

លក្ខណៈបច្ចេកទេសរបស់ឧបករណ៍ ពីលំដាប់សំខាន់បំផុត (1) ចុះមកលំដាប់សំខាន់តិចតួច (5):

1)……………………………………………………………………………………………………
2)……………………………………………………………………………………………………
3)……………………………………………………………………………………………………
4)……………………………………………………………………………………………………
5)……………………………………………………………………………………………………

តេស្តដែលប្រើដើម្បីដំណើរការ:

1)……………………………………………………………………………………………………
2)……………………………………………………………………………………………………
3)……………………………………………………………………………………………………
4)……………………………………………………………………………………………………
5)……………………………………………………………………………………………………

ចូរគូរគំនូរព្រាងនៅទីនេះ ពីរចនានៃឧបករណ៍របស់អ្នក! ដាក់ស្លាកវិមាត្រ និងទំហំ; បញ្ជាក់ថាតើ វាជាឧបករណ៍យួរដៃ អាចដឹកជញ្ជូនបានឬទេ និងប៉ាន់ស្មានតម្លៃដើមក្នុងការទិញ និងតម្លៃពេលប្រតិបត្តិការ។

……
……………………………………………………………………………
……
……
……
……
……
…………………………………………………………………………………………………
……………………………………………

ប្រធានបទទី8: គោលការណ៍ណែនាំ និងគោលនយោបាយថ្នាក់ជាតិ នៃការធ្វើតេស្តជិតអ្នកជំងឺ។

តើប្រទេសកម្ពុជាមានគោលការណ៍ណែនាំ និងគោលនយោបាយស្តីពីការធ្វើតេស្តជំងឺកូវីដ-19ដែលស្ថិតនៅជិតអ្នកជំងឺដែរឬទេ?

តើមានអ្នកណាដែលកំពុងសរសេរឯកសារបែបនេះសម្រាប់ប្រទេសកម្ពុជាដែរឬទេ?

ឧបមាថាយើងកំពុងធ្វើសេចក្តីព្រាងឯកសារនេះ។ សូមសរសេរគ្រោងនៃឯកសារនេះ ដូចខាងក្រោម:

ចម្លើយ:

តួនាទីនៃគោលការណ៍ណែនាំ

និងគោលនយោបាយស្តីពីការធ្វើតេស្តជិតអ្នកជំងឺក្នុងវិបត្តិសេដ្ឋកិច្ចដោយសារ ជំងឺកូវីដ-19៖

………
………
…….

គោលបំណងនៃគោលការណ៍ណែនាំ និងគោលនយោបាយស្តីពីការធ្វើតេស្ត៍ជិតអ្នកជំងឺ

1)……………………………………………………………………………………………………
2)……………………………………………………………………………………………………
3)……………………………………………………………………………………………………

តើមានអ្នកណាកំពុងសរសេរឯកសារបែបនេះសម្រាប់ប្រទេសកម្ពុជាដែរឬទេ? បើមាន តើជានរណាគេ?

………
………
…….

Theory. PV GM^2 graphs help compare tiered sensitivity and specificity, government-authorized tests, commercial claims, and clinically evaluated rapid antigen tests (RAgTs). Tiered sensitivity/specificity comprise: T1) 90%, 95%; T2) 95%, 97.5%; and T3) 100%, ≥99%, respectively. Refinements and improvements in sensitivity and specificity help customize tests for clinical objectives. PV GM^2 pattern recognition reveals the significance of uncertainty when also considering 95% confidence intervals. PV GM^2 is not intended for point comparisons. Instead, please compare the performance configurations ("fingerprints") of the PV GM^2 curves.

COVID-19 Rapid Antigen Tests in Cambodia. Two point-of-care formats became available in Southeast Asia in 2021, the Roche Diagnostics SARS-CoV-2 Rapid Antigen Test and the Abbott Panbio COVID-19 Antigen Test, for which **Table S1** and **Table S2** present detailed summaries of performance from clinical evaluations, metastudies, and manufacturer claims. These tables provide the data for mathematical analysis using the equations in **Table S3**.

Figure S1. Roche Diagnostics Rapid Antigen Test Performance [2021]

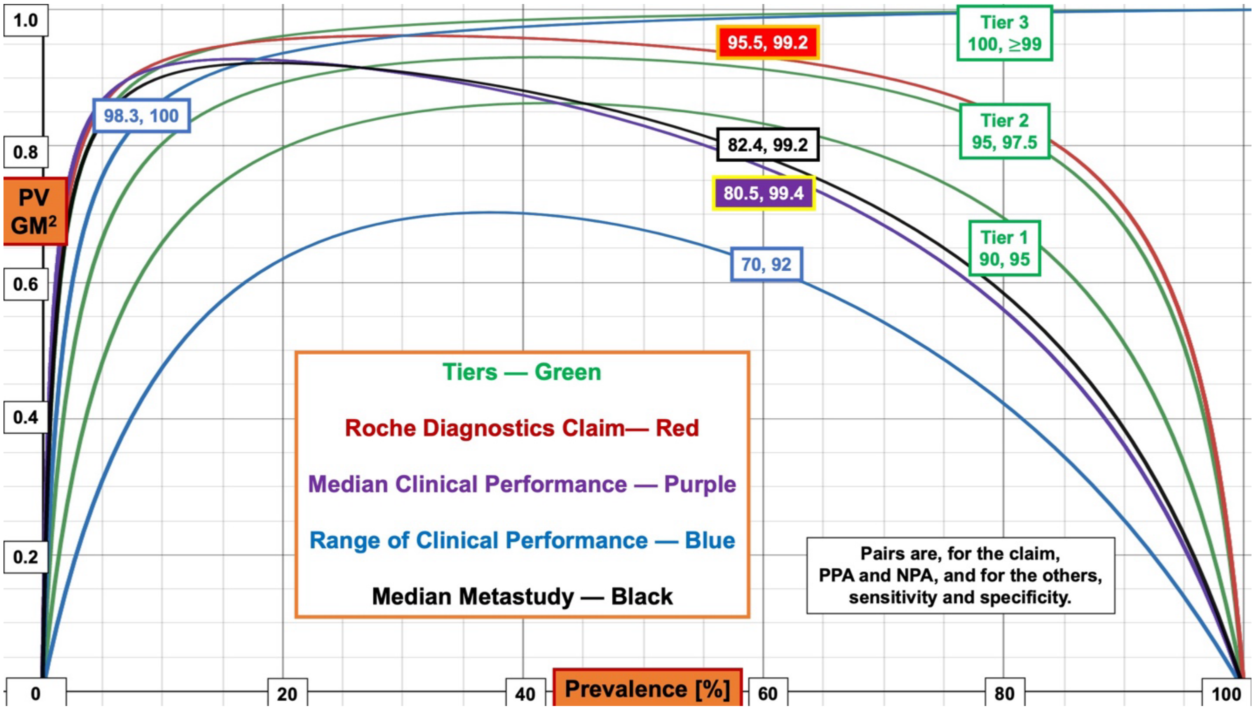

Figure S1 presents detailed visual logistics for the SARS-CoV-2 Rapid Antigen Test (Roche Diagnostics). The PV GM^2 curves fall-off rapidly with increasing prevalence typical of the poor clinical sensitivity of COVID-19 point-of-care antigen tests. Boxed pairs in **Figure S1** identify sensitivity and specificity or positive (PPA) and negative percent agreement (NPA) in percent.

The blue curves bracket the highest and lowest performance demonstrated in the clinical evaluations listed in **Table S1**. The purple line represents the median clinical performance from those evaluations. The clinical median lies remarkably close to the black curve reflecting the median of metastudies. Both exceed the WHO threshold of 80% for sensitivity, but the WHO

threshold is too low to reliably rule out COVID-19 as prevalence increases. Rapid antigen tests with poor sensitivity generate false negatives, increasingly so as prevalence increases.

The median clinical performance of the Roche Diagnostics RAgT does not match the manufacturer claim (red curve). However, the assay is somewhat balanced in that it displays a relatively "flat top" of the PV GM^2 curve in the low range of prevalence up to 40%. The manufacturer claims of PPA and NPA equal to 95.5% and 99.2% exceed Tier 2 thresholds for sensitivity and specificity of 95% and 97.5%, respectfully. However, in real-world clinical evaluations the median clinical performance of the Roche Diagnostics RAgT exceeds the Tier 2 curve only when prevalence is low. Thus, this RAgT will generate false negative results as the prevalence increases.

Table S1. Roche Rapid Antigen Test Clinical Evaluations [2021]

**Author/Organ., Journal, Year**[1]	**Sensitivity (CI), %**	**Specificity (CI), %**	**Sensitivity (CI), % @ Cycle Threshold, Ct ≤ x or range**	**Sample Size**
Nalumansi	**70** (NR)	**92** (87-96)	92 Ct ≤ 29	262
Krüttgen	**70.7** (NR)	**96** (NR)	100% Ct < 25	150
Cerutti	**72.1** (83.69-93.06)	**100** (98.36-100)	100 (NR), Ct ≤ 28	330
Salvagno	**72.5** (NR)	**99.4** (NR)	97-100% Ct < 25	321
Lindner 2020	**74.4** (58.9-85.4)	**99.6** (97.8-100)	96.2 Ct 17.3-25.3	289
Krueger	**76.6** (62.8-86.4)	**99.3** (98.6-99.6)	100 (82.4-100), Ct ≤ 25	1,263
Lindner 2021	**80.5** (nasal), 73.2 (NP)	**98.6** (94.9-99.6)	100 (Nasal), 94.7 (NP); > 7.0 log10 RNA SARS-CoV2/swab	179
Favresse	**82.5** (NR) (Ct < 33)	**100** (all Ct) 91.5 (Ct < 25)	96.6% Ct < 25	188
Igloi	**84.9** (79.1-89.4)	**99.5** (98.7-99.8)	99.1 (95.2-100) Ct < 25	970
FIND Report	**84.97** (78.3-90.23)	**98.94** (98.23-99.39)	97.14 (90.1-99.65), Ct ≤ 25	1,659
Hospital Universitaires Genève	**89.0** (83.69-93.06)	**99.70** (98.36-99.99)	98 (NR), Ct ≤ 22	529
Schwob	**92.9** (86.4-96.9)	**100** (NR)	96.6 (90.5-99.3) Ct ≤ 26	982
Chaimayo	**98.3** (91.06–99.96)	**98.7** (97.06–99.59)	NR	454
**Statistical Summary**				
**Median** [N = 13 studies] [Range]	**80.5** [70-98.3]	**99.4** [92-100]		
**Metastudies & Summary**				**Notes**
**Brummer**	**81.7** [74.8-87.0]	**99.2** [97.0-99.8]		Pooled specificity
**Hayer**	**82.4** [74.2-88.4]	**99.6** [99.0-99.8]		Spec. from Dinnes
**Dinnes**	**88.1** [84.2-91.1] 69.2 [28.6-90.9]	**99.1** [97.8-99.6] 99.1 [95.2-100]		Symptomatic Asymptomaic
**Median**	**82.4** [81.7-88.1]	**99.2** [99.1-99.6]		
**Manufacturer Claim**				

**Roche Diagnostics** *Quick Fact Sheet*[2] *& Test Specifications*[3] 2021	91.2 (86.9 – 94.4) [not claimed]	**99.2** (98.2 – 99.7)	**95.5** (91.8 – 97.8), Ct ≤ 30	Specificity, N = 727 Sensitivity, N = 249 Ct ≤ 30 , N = 220

Notes: 1. Medians refer to sensitivities. 2. The Roche Diagnostics COVID-19 rapid antigen test does not have US Food and Drug Administration Emergency Use Authorization status as of June 2021. 3. Studies with no or limited sensitivity evaluation based on Ct values available were excluded.

Abbreviations: CI, 95% confidence interval; and NR, not reported or not applicable.

References (to be inserted in the list of references and the numbering harmonized—generally, do not change the format or delete content)

1. Roche Diagnostics. *SARS-CoV-2 Rapid Antigen Test. Primary and secondary influencers on assay performance.* Update May 28, 2021. [Consult document for primary references listed in table by the first author's last name.] https://www.bing.com/search?form=MOZLBR&pc=MOZI&q=SARS-CoV-2-Rapid-Antigen-Assay-Clinical-Performance-2021.05.pdf [Accessed June 15, 2021.]

2. Roche Diagnostics Thailand. *Quick Fact Sheet SARS-CoV-2 Rapid Antigen Test* - MC-TH-00488. June 2021.

3. Roche Diagnostics. *SARS-CoV-2 Rapid Antigen Test. Test Specifications.* https://diagnostics.roche.com/global/en/products/params/sars-cov-2-rapid-antigen-test.html#productSpecs [Accessed June 15, 2021.]

Figure S2. PanBio Rapid Antigen Test Performance [2021]

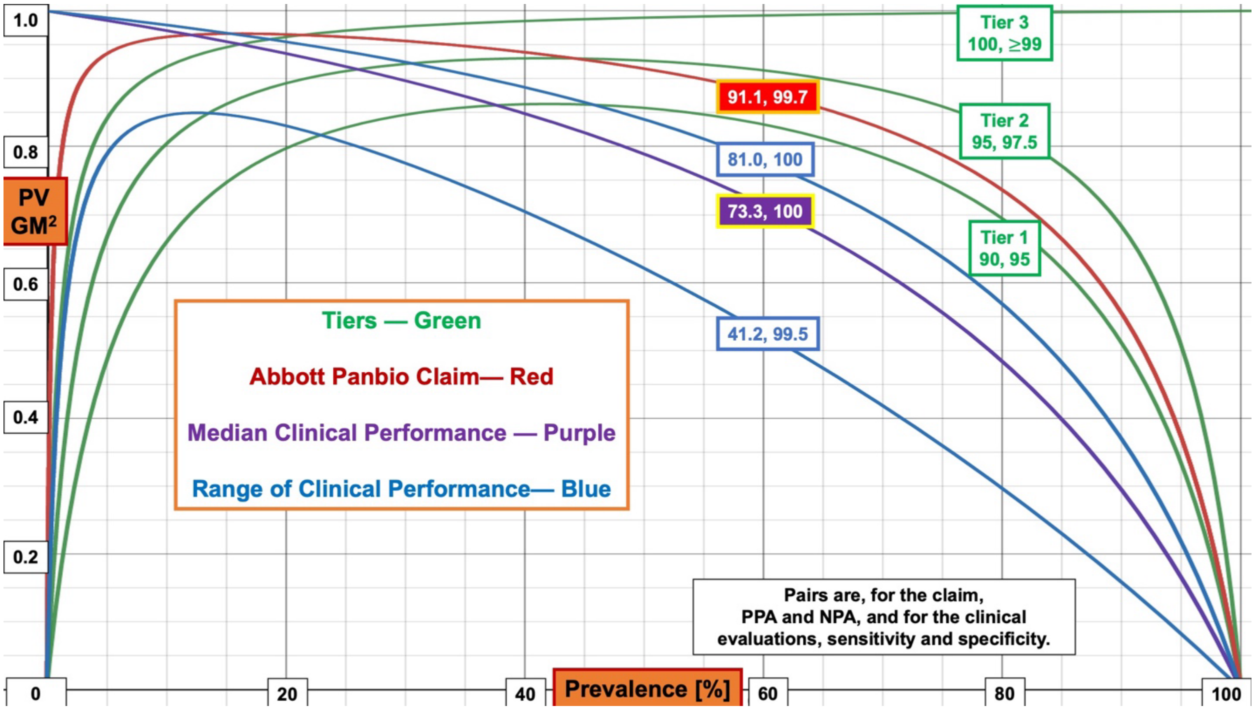

Figure S2 presents analysis of the Panbio COVID-19 antigen test (Abbott) based on the clinical evaluations in **Table S2**. Fall-off in clinical performance as prevalence increases is steep with a median that breaks below Tier 2 at very low prevalence, then falls precipitously. Hence,

false negatives will increase exponentially as prevalence increases. In clinical evaluations the vertical range between high and low performance is somewhat limited but also positioned lower than that in **Figure S1**. The manufacturer claim of performance (red curve) does not achieve Tier 2 performance above 44.2% prevalence. The median clinical performance of the Panbio COVID-19 antigen test does not achieve the WHO threshold for sensitivity of 80%.

Table S2. Panbio Rapid Antigen Test Clinical Evaluations [2021]

Author, Journal, Year	Sensitivity [CI], %	Specificity [CI], %	Clinical Details, Time Interval, Symptoms, Sample Size, POC	Technical Notes
**Clinical Evaluations**				
**Caruana** *Microorgan* 2021	**41.2** [NR]	**99.5** [NR]	Emergency ward of a Swiss University Hospital. N = 532.	Sensitivity (69.2%) higher for symptomatic patients within 4 days.
**Villaverde** *J Peds* 2021	**45.5** [34.1-57.2]	**99.8** [99.4-99.9]	Symptomatic pediatric patients ages 1-16 years presenting within 5 days of symptom onset to the emergency departments of 7 centers. N = 1620	Concordance between RT-PCR and Ag test: *kappa* = 0.6. High false-negative Ag tests (54.5%) may have public health implications.
**Torres** *Clin Micro Infect* 2020	**48.1** [37.4-58.9]	**100** [99.3-100]	Asymptomatic household and non-household contacts of COVID-19 cases. POC study. Higher sensitivity in household subjects, more likely to become symptomatic. N = 614.	Median time to test 7 days [1-7 range].
**Masia** *Open Forum Infect Dis* 2021	**60.5** [53.5-67.4]	**100** [99.3-100]	Three primary care centers and an emergency department. POC study. N = 913.	Highest sensitivity with NP swabs. Higher sensitivity (80%) for Ct < 30.
**Bulilete** *J Infect* 2021	**71.4** [63.1-78.7]	**99.8** [99.4-99.9]	Symptomatic patients and close contacts in primary healthcare centers and test sites. N = 1,369.	Most tested within 5 days of symptom onset. Higher sensitivity (86.2%) when Ct <25.
**Nordgren** *J Clin Virol* 2021	**71.8** [64.0-78.7]	**100** [97.2-100]	Patients with respiratory and/or COVID-19 symptoms. N = 286.	Stored samples tested within 1 week median 2 days). Ct > 40 excluded.
**Linares** *J Clin Virol* 2020	**73.3** [62.2-83.8]	**100** [NR]	Emergency department and primary healthcare centers. 72.1% of patients were symptomatic. N = 255.	86.5% sensitivity, first week, symptomatic patients. Median Ct 23.
**Landaas** *J Clin Virol* 2021	**74.4** [69-79]	**99.9** [99.7-99.9]	Prospective study. Test station in Oslo, and from COVID-19 outbreaks in six Norwegian municipalities. N =4,857.	For Ct < 30, 83.8% sensitivity. Lower (55.3%) in asymptomatic persons.
**Soleimani** *J Med Virol* 2021	**75.0** [68.9-80.4]	**100** [97.8-100.0]	Non-complicated suspected COVID-19 subjects. N = 401. NP swabs.	Sensitivity (88.8%) greater for Ct < 30.
**Gonzalez-Donapetry** *Ped Infect Dis* 2021	**77.78** [51.92-92.63]	**100** [98.88-100]	Pediatric population in the emergency department. N = 440. Authors state sensitivity is lower in children. Also, must confirm Ag negative results.	All Ag positive result had Ct < 34 by RT-PCR.
**Albert** *Clin Micro Infect*	**79.6** [67.0-88.8]	**100** [98.7-100]	Symptomatic patients. POC study in primary healthcare centers. N = 412.	Claims to be first POC study. Could not detect

2021				virus in RT-PCR +/Ag – specimens.
**Gremmels** *E Clin Med* 2021	72.6 [64.5-79.9] **81.0** [69.0-89.8]	**100** [99.7-100]	Community-dwelling and mildly symptomatic subjects. Sensitivity reported for N = 1,367 & 208 in 2 decentralized sites, respectively.	Sensitivities higher if Ct < 32. Short TAT, low cost, and user friendly. High prevalence.
**Statistical Summary**				
**Median [N = 13]** [Range]	**73.3** [41.2-81.0]	**100** [99.5-100]	All clinical studies listed above.	
**Manufacturer Claim**				
**Abbott Panbio** *Brochure Global* 2021	**91.1** [84.2-95.6]	**99.7** NPA [98.6-100]	Individuals suspected of exposure within 7 days. Nasal swabs. N not reported. Immunochromatographic antigen detection of SARS-CoV-2. FDA EUA RT-PCR reference.	Claimed sensitivity of 99.0% [CI NR] for Ct ≤ 33. Above 33, "not infectious."

Notes: 1. Median refers to sensitivities. 2. The Panbio COVID-19 rapid antigen detection test does not have US Food and Drug Administration Emergency Use Authorization status as of June 13, 2021.

Abbreviations: Ag, antigen; CI, 95% confidence interval; COVID-19, Coronavirus Disease-19; Ct, cycle threshold; EUA, Emergency Use Authorization; FDA, Food and Drug Administration (USA); N, sample size; NP, nasopharynge4al; NPA, negative percent agreement; NR, not reported; POC, point-of-care; PPA, positive percent agreement; RT-PCR, reverse transcription-polymerase chain reaction; and TAT, turnaround time.

References

1. Caruana G, Croxatto A, Kampouri E, Kritikos A, Opota O, Foerster M, et al. Implementing SARS-CoV-2 Rapid Antigen Testing in the Emergency Ward of a Swiss University Hospital: The INCREASE Study. *Microorganisms.* 2021 Apr 10;9(4):798. doi: 10.3390/microorganisms9040798.

2. Villaverde S, Domínguez-Rodríguez S, Sabrido G, Pérez-Jorge C, Plata M, Romero MP, et al. Epidemiological Study of COVID-19 in Children of the Spanish Society of Pediatric (EPICO-AEP) Working Group. Diagnostic Accuracy of the Panbio Severe Acute Respiratory Syndrome Coronavirus 2 Antigen Rapid Test Compared with Reverse-Transcriptase Polymerase Chain Reaction Testing of Nasopharyngeal Samples in the Pediatric Population. *J Pediatr.* 2021 May;232:287-289.e4. doi: 10.1016/j.jpeds.2021.01.027

3. Torres I, Poujois S, Albert E, Colomina J, Navarro D. Evaluation of a rapid antigen test (Panbio™ COVID-19 Ag rapid test device) for SARS-CoV-2 detection in asymptomatic close contacts of COVID-19 patients. *Clin Microbiol Infect.* 2021Apr;27(4):636.e1-636.e4. doi: 10.1016/j.cmi.2020.12.022

4. Masiá M, Fernández-González M, Sánchez M, Carvajal M, García JA, Gonzalo-Jiménez N, et al. Nasopharyngeal Panbio COVID-19 Antigen Performed at Point-of-Care Has a High Sensitivity in Symptomatic and Asymptomatic Patients With Higher Risk for Transmission and Older Age. *Open Forum Infect Dis.* 2021 Feb 2;8(3):ofab059. doi: 10.1093/ofid/ofab059

5. Bulilete O, Lorente P, Leiva A, Carandell E, Oliver A, Rojo E, et al; COVID-19 Primary Care Research Group. Panbio™ rapid antigen test for SARS-CoV-2 has acceptable accuracy in symptomatic patients in primary health care. *J Infect*. 2021 Mar;82(3):391-398. doi: 10.1016/j.jinf.2021.02.014

6. Nordgren J, Sharma S, Olsson H, Jämtberg M, Falkeborn T, Svensson L, et al.. SARS-CoV-2 rapid antigen test: High sensitivity to detect infectious virus. *J Clin Virol.* 2021 Apr 24;140:104846. doi: 10.1016/j.jcv.2021.104846

7. Linares M, Pérez-Tanoira R, Carrero A, Romanyk J, Pérez-García F, Gómez-Herruz P, et al. Panbio antigen rapid test is reliable to diagnose SARS-CoV-2 infection in the first 7 days after the onset of symptoms. *J Clin Virol.* 2020 Dec;133:104659. doi: 10.1016/j.jcv.2020.104659

8. Landaas ET, Storm ML, Tollånes MC, Barlinn R, Kran AB, Bragstad K, et al. Diagnostic performance of a SARS-CoV-2 rapid antigen test in a large, Norwegian cohort. J Clin Virol. 2021 Apr;137:104789. doi: 10.1016/j.jcv.2021.104789

9. Soleimani R, Deckers C, Huang TD, Bogaerts P, Evrard S, Wallemme I, et al. Rapid COVID-19 antigenic tests: Usefulness of a modified method for diagnosis. *J Med Virol*. 2021 May 19. doi: 10.1002/jmv.27094

10. González-Donapetry P, García-Clemente P, Bloise I, García-Sánchez C, Sánchez-Castellano MÁ, Romero MP, et al. SARS-CoV-2 Working Group. Think of the Children: Evaluation of SARS-CoV-2 Rapid Antigen Test in Pediatric Population. *Pediatr Infect Dis J*. 2021 May 1;40(5):385-388. doi: 10.1097/INF.0000000000003101

11. Albert E, Torres I, Bueno F, Huntley D, Molla E, Fernández-Fuentes MÁ, et al. Field evaluation of a rapid antigen test (Panbio™ COVID-19 Ag Rapid Test Device) for COVID-19 diagnosis in primary healthcare centres. *Clin Microbiol Infect*. 2021 Mar;27(3):472.e7-472.e10. doi: 10.1016/j.cmi.2020.11.004

12. Gremmels H, Winkel BMF, Schuurman R, Rosingh A, Rigter NAM, Rodriguez O, et al. Real-life validation of the Panbio™ COVID-19 antigen rapid test (Abbott) in community-dwelling subjects with symptoms of potential SARS-CoV-2 infection. *E Clinical Medicine*. 2021 Jan;31:100677. doi: 10.1016/j.eclinm.2020.100677

13. Abbott Panbio. *Panbio COVID-19 Ag Nasal Swab Brochure Global.* Panbio COVID19 Ag Rapid Test Device. For people suspected of COVID-19 exposure. [Not approved for sale in the United States.] https://content.veeabb.com/1d09429b-8373-419f-8f1a-d28f9586863a/da32877d-2963-4b16-913e-b5b806a559b9/da32877d-2963-4b16-913e-b5b806a559b9_source__v.pdf [Accessed June 14, 2021.]

CONCLUSIONS REGARDING COVID-19 TEST PERFORMANCE

• Generally, point-of-care RAgTs work best when viral load is high in the early phase of symptom onset, that is, fewer than 5-7 days. However, caution is in order because in real-world settings RAgTs typically display performance that is inferior to manufacturer claims.

• The Roche Diagnostics SARS-CoV-2 Rapid Antigen Test [2021] performed reasonably well at low prevalence while being deemed an assay achieving the WHO criterion for sensitivity of 80% (or greater) based on median clinical performance documented in several studies confirming or ruling out COVID-19 in people with signs and symptoms of acute infection.

• Rapid antigen tests of asymptomatic patients generally do not perform well. Please see the references below for detailed analysis of RAgTs, comparison to molecular diagnostics (including home LA MP tests), and discussion of repeat testing protocols.

• Best clinical use of RAgTs will be achieved thorough education and training in preanalytic, analytic, and postanalytic testing processes defined by each manufacturer with exact timed swabbing procedures performed when symptoms and viral load peak.

• Inferior specimen procurement or processing, as well as unacceptable environmental conditions, can decrease sensitivity, increase false negatives, and send people forward believing they are free of disease, when in fact, they unknowingly spread COVID-19 and its variants.

Table S3. Mathematical Equations —

Fundamental Definitions, Derived Equations, Ratios, Rates, Predictive Value Geometric Mean-squared, Prevalence Boundary, Recursion, and Special Cases

Eq. No.	Category and Equations	Dep. Var.	Indep. Var.
	**Fundamental Definitions**		
1	x = Sens = TP/(TP + FN)	x	TP, FN
2	y = Spec = TN/(TN + FP)	y	TN, FP
3	s = PPV = TP/(TP + FP)	s	TP, FP
4	t = NPV = TN/(TN + FN)	t	TN, FN
5	p = Prev = (TP + FN)/N	p	TP, FN, N
6	N = TP + FP + TN + FN	N	TP, FP, TN, FN
	**Derived Equations**		
7	PPV = [Sens•Prev]/[Sens•Prev + (1-Spec)(1-Prev)], or s = [xp]/[xp + (1-y)(1-p)] — symbolic version of the equation above	s	x, y, p
8	p = [s(y-1)]/[s(x + y - 1) - x]	p	x, y, s
9	x = [s(p-1)(y-1)]/[p(s-1)]	x	y, p, s
10	y = [sp(x-1) + s - px]/[s(1-p)]	y	x, p, s
11	NPV = [Spec•(1-Prev)]/[Prev•(1–Sens) + Spec•(1–Prev)], or t = [y(1–p)]/[p(1–x) + y(1–p)]	t	x, y, p
12	p = [y(1-t)]/[t(1 - x - y) + y]	p	x, y, t
13	x = [pt + y(1-p)(t-1)]/[pt]	x	y, p, t
14	y = [pt(x-1)]/[t(1-p) – 1 +p]	y	x, p, t
	**Ratios**		
15	TP/FP = PPV/(1-PPV) = [Sens•Prev]/[(1-Spec)(1-Prev)], or [xp]/[(1-y)(1-p)]	TP/FP Ratio	x, y, p
16	FP/TP = (1-PPV)/PPV = [(1-y)(1-p)]/(xp)	FP/TP Ratio	x, y, p
17	FN/TN = (1-NPV)/NPV = [p(1-x)]/[y(1-p)]	FN/TN Ratio	x, y, p
	**Rates**		
	True positive (R_{TP}), false positive (R_{FP}), & positive (R_{POS})		
18	R_{TP} = TP/(TP + FN) = x	R_{TP}	TP, FN
19	R_{FP} = FP/(TN + FP) = 1 – Spec = 1- y	R_{FP}	TN, FP
20	R_{POS} = (TP + FP)/N	R_{POS}	TP, FP, N
	False Omission (R_{FO})		
21	R_{FO} = FN/(TN + FN) = 1 – NPV = 1- t = [p(1-x)]/[p(1-x) + y(1-p)]	R_{FO}	x, y, p
	R_{FO} with repeated test (rt)		
22	$R_{FO/rt} = [p(1-x)^2]/[p(1-x)^2 + y^2(1-p)]$	$R_{FO/rt}$	x, y, p

	**Predictive value geometric mean-squared (range 0 to 1)**		
23	PV GM^2 = PPV•NPV = s•t = {[xp]/[xp + (1-y)(1-p)]}• {[y(1–p)]/[p(1–x) + y(1–p)]}	PV GM^2	x, y, p
	**Prevalence Boundary**		
	Prevalence boundary for one test given R_{FO}		
24	PB = y(1-t)/[(1-x) - (1-t)(1-x-y)] = [yR_{FO}]/[(1-x) -R_{FO}(1-x-y)] = [yR_{FO}]/[R_{FO}(x+y-1) + (1-x)]	PB	x, y, t or x, y, R_{FO}
	Prevalence boundary for repeated test (PB_{rt}) given R_{FO}		
25	PB_{rt} = [y^2R_{FO}]/[R_{FO}(y^2-x^2+2x-1) + $(x-1)^2$]	PB_{rt}	x, y, R_{FO}
	Improvement in prevalence boundary (ΔPB) when test second time given R_{FO}		
26	ΔPB = {y^2R_{FO}/[R_{FO}(y^2-x^2+2x-1) + $(x-1)^2$]}-{yR_{FO}/[R_{FO}/[(x+y-1) + (1-x)]}	ΔPB	x, y, R_{FO}
	**Recursion**		
	Recursive formulae for PPV (s_{i+1}) and NPV (t_{i+1})		
27	s_{i+1} = [xp_i]/[xp_i + (1-y)(1-p_i)], where the index, i = 1, 2, 3…	s_{i+1}	x, y, p_i
28	t_{i+1} = [y(1-p_i)]/[p_i(1-x) + y(1-p_i)]	t_{i+1}	x, y, p_i
	**Special Cases**		
	PPV when sensitivity is 100%		
29	PPV = [Prev]/[Prev + (1-Spec)•(1-Prev)], or s = [p]/[p + (1-y)(1-p)]	s	y, p
	Prevalence when sensitivity is 100% (i.e., FN = 0)		
30	Prev = 1 – [(1 – N_+/N)/Spec], or p = 1- [(1-$POS_{\%}$)/y]	p	$POS_{\%}$, y
	Sensitivity when given specificity, R_{FO}, and PB (no repeat)		
31	x = [PB-R_{FO}(y+PB-y•PB)]/[PB(1-R_{FO})]	x	y, R_{FO}, PB
	Sensitivity, given R_{FO} and PB, when specificity (y) is 100%		
32	x = (PB-R_{FO})/[PB(1-R_{FO})]	x	R_{FO}, PB
	Accuracy (not recommended – see note)		
33	A = (TP + TN)/N = Sens•Prev(dz) + Spec•Prev(no dz)	A	TP, TN, N

Abbreviations

Dep. Var., dependent variable; Eq., equation; FN, false negative; FP, false positive; i, an index from 1 to 3 or more (the number of testing events); Indep. Var., independent variable(s); N, total number of people tested; N_+, number of positives (TP + FP) in the tested population; N-, number of negatives (TN + FN) in the tested population; $NEG_{\%}$, (N-/N), percent negative of total number tested; NPV, negative predictive value (t); p_{i+1}, p_i, indexed partition prevalence in the recursive formula for PPV and NPV; PB, prevalence boundary; PB_{rt}, prevalence boundary for repeated test; ΔPB, improvement in prevalence boundary; $POS_{\%}$, (N_+/N), percent positive of the total number tested (same as R_{POS}); PPV, positive predictive value (s); Prev, prevalence (p); Prev(dz), same as p; Prev(no dz), prevalence of no disease; PV GM^2, square of the geometric mean of positive and negative predictive values, (PPV•NPV), expressed as a fraction from 0 to 1; R_{FO}, the rate of false omissions; $R_{FO/rt}$, rate of false omission with repeated test (rt); R_{FP}, false positive rate, aka false positive alarm (probability that a false alarm will be raised or that a false result will be reported when the true value is negative); R_{POS}, positivity rate; R_{TP}, true positive rate, the same as sensitivity; Sens, sensitivity (x); Spec, specificity (y); TN, true negative; and TP, true positive.

Notes

Sens, Spec, PPV, NPV, and Prev are expressed as percentages from 1 to 100%, or as decimal fractions from 0 to 1 by dividing by 100%.

PV GM^2 was created for visual logistics comparisons of performance curves of diagnostic tests, not for point comparisons.
If the denominators of derived equations become indeterminate, then revert to the fundamental definitions, Eqs. 1-6.
The use of the formula for accuracy [Eq. 33] is not recommended, because of duplicity of values with complementary changes in sensitivity and specificity.
Equation Set Version 27.0 • August 27, 2023

Table S4. COVID-19 Tests Performed in the Mobile Hybrid Laboratory-POCT Van for Rural Communities during the Pandemic in Northern California

Time to Result (min) FDA Status	Instrument/Test Manufacturer	Speci -men	Storage/ Testing Temperatures (°C), Details	Sensitivity (%) Specificity (%)
COVID-19 Tests *(in order of time required to produce a result)*				
**10**<br>FDA EUA 142916	CareStart COVID-19 Antigen Test<br>Access Bio, Inc.	ANS<br>NS	1~30/15-30<br>Lateral Flow, AT, Visually Read	87.18, 100 (AN); 93.75, 99.32 (NP)
**<15**<br>FDA EUA 139789	COVID-19 IgG/IgM Rapid Test<br>Assure Tech (Hangzhou Co., Ltd)	WB<br>Pl<br>S	2~30/15-30<br>Lateral Flow Chromatographic Immunoassay, AT, Visually Read	88.7, 93.3
**15**<br>FDA EUA 137886	Sofia SARS Antigen Fluorescent Immunoassay<br>Quidel Corporation	NS	15-35, Immuno-fluorescence-based Lateral Flow, AT, Instrument Read	96.7, 100
**15**<br>FDA EUA 142701	Sofia 2 Flu + SARS Antigen Combo Fluorescent Immunoassay<br>Quidel Corporation	NS<br>NPS	15-35 Immuno-fluorescence-based Lateral Flow, AT, Instrument Read	Flu A (NS): 90, 95; Flu A (NP): 97.1, 94.6; Flu B (NS): 89, 96; Flu B (NP) 90, 97; SARS-CoV-2: 95.2, 100
**20**<br>FDA EUA 151212	INDICAID COVID-19 Rapid Antigen Test<br>PHASE Scientific International Ltd.	NS<br>NPS	2-30/15-30 Lateral Flow Immunoassay, AT, Visually Read	84.4,96.3
**<30**<br>FDA EUA 136345	Accula SARS-CoV-2 Test<br>Mesa Biotech, Inc.	NS<br>NMT<br>S	15-30/15-30<br>RT-PCR & Lateral Flow Immuno-assay, Cartridge-based, Visually Read	95.8, 100
**<30**<br>FDA EUA 136522	ID NOW COVID-19<br>Abbott Diagnostics Scarborough, Inc.	NS<br>NPS<br>TS	2-30/15- 30<br>Isothermal Nucleic Acid Amplifica-tion, Cartridge-based, Machine Read	95.0, 97.9
**30**<br>FDA EUA 144253	Clip COVID-19 Rapid Antigen Test<br>Luminostics, Inc.	ANS	15- 30/15- 30<br>Lateral Flow Immuno-luminescent, AT, Machine Read	96.9, 100

Other Infectious Disease Tests				
**5**<br>CLIA Waived<br>1127458/McKesson-Brand-181-34125	Consult Diagnostics Strep A<br>McKesson Medical-Surgical Inc.	TS	15-30/15-30<br>Immunoassay Ag Test Cassette, Visually Read	96.2, 98.7
**<15**<br>CLIA Waived<br>1076728/McKesson-Brand-181-36025	Consult Influenza A & B Test<br>McKesson Medical-Surgical Inc.<br>[Princeton BioMeditech Corp.]	NS<br>NPS	2-30 /18-30<br>AT, Visually Read	Flu A (NS): 91.7, 75.2 Flu B (NS): 82.4, 88.3; Flu A (NP): 89.6, 77 Flu B (NP): 86.8, 92.9
Specimen Collection *(for off-site testing)*				
**>24 hr**[a]<br>pdf20/DEN200031	BioFire Respiratory Panel 2.1<br>BioMérieux, Inc.	NPS	15-25 (4 hours), 2-8 (3 days), ≤-15 ≤-70 (30 days)/15-25<br>PCR-based multi-plex, Machine Read	97.1, 99.3

Abbreviations: ANS, anterior nasal swab; AT, antigen test; CB, capillary blood (lancet fingerstick); CBC, complete blood count; CE, Conformité Européenne; CLIA, Clinical Laboratory Improvement Amendments; COVID-19, Coronavirus disease 2019; EUA, Emergency Use Authorization; FDA, United States Food and Drug Administration; Flu, influenza; IgG, immunoglobulin G; IgM, immunoglobulin M; NMTS, nasal mid-turbinate swab; NPS, nasopharyngeal; NS, nasal swab; RT-PCR, reverse-transcriptase polymerase chain reaction; Pl, plasma; S, serum; SARS-CoV-2, severe acute respiratory syndrome Coronavirus 2; TS, throat swab; TSH, thyroid stimulating hormone; and WB, whole blood.

Footnote: a) The specimen collected is shipped overnight to a third-party laboratory to be processed on a BioFire® FilmArray 2.0 system. The analysis time is 45 minutes.

REFERENCES — THE MATHEMATICS OF COVID-19 DIAGNOSTIC TESTS

Kost GJ. The impact of repeating COVID-19 rapid antigen tests on prevalence boundary performance and missed diagnoses. Diagnostics 2023;13(3223):1-14. [open access]

Kost GJ. Changing diagnostic culture calls for point-of-care preparedness — multiplex now, open prevalence boundaries, and build community resilience. Journal of 21st Century Pathology 2022;2(5/129):1-7. [open access]

Kost GJ. The Coronavirus Disease 2019 spatial care path: Home, community, and emergency diagnostic portals. Diagnostics 2022;12 (1216): 1-16. [open access]

Kost GJ. The COVID-19 Grand Challenge: Setting expectations and future directions for community and home testing. Archives of Pathology & Laboratory Medicine (APLM) 2022;146:789-790. [open access]

Kost GJ. Home antigen test recall affects millions: Beware false positives, but also uncertainty and potential false negatives. APLM 2021;146:403. [open access]

Kost GJ. Diagnostic strategies for endemic Coronavirus disease 2019 (COVID-19) — Rapid antigen tests, repeated testing, and prevalence boundaries. APLM 2021;146:16-25. [open access]

Kost GJ. Public health education should include point-of-care testing: Lessons learned from the COVID-19 pandemic. e-Journal of the International Federation of Clinical Chemistry and Laboratory Medicine 2021:32(3):311-327. [open access]

Eng M, Zadran A, Kost GJ. COVID-19 risk avoidance and management in limited-resource countries. Point-of-care strategies for Cambodia. Omnia Digital Health 2021;6/7:112-115. [open access]

Kost GJ. The impact of increasing disease prevalence, false omissions, and diagnostic uncertainty on Coronavirus disease 2019 (COVID-19) test performance. APLM 2021;145:797-813. [open access]

Kost GJ. Designing and interpreting COVID-19 diagnostics: Mathematics, visual logistics, and low prevalence. APLM 2021;145:291-307. [open access]

Kost GJ. Geospatial spread of antimicrobial resistance, bacterial and fungal threats to COVID-19 survival, and point-of-care solutions. APLM 2021;145:145-167. [open access]

Kost GJ. Geospatial hotspots need point-of-care strategies to help stop highly infectious outbreaks: Ebola and Coronavirus-19. APLM 2020;144:1166-1190. [open access]

Supplement Version F7.0 • June 20, 2024

END OF SUPPLEMENT
